# Osteocytes Enhance Osteogenesis by Autophagy-Mediated FGF23 Secretion Under Mechanical Tension

**DOI:** 10.3389/fcell.2021.782736

**Published:** 2022-01-31

**Authors:** Huiyue Xu, Meng Xia, Lian Sun, Hua Wang, Wei-Bing Zhang

**Affiliations:** ^1^ Jiangsu Key Laboratory of Oral Diseases, Nanjing Medical University, Nanjing, China; ^2^ Department of Orthodontics, Affiliated Hospital of Stomatology, Nanjing Medical University, Nanjing, China; ^3^ Department of Stomatology, Dushu Lake Hospital Affiliated to Soochow University, Suzhou, China; ^4^ Department of Stomatology, Medical Center of Soochow University, Suzhou, China

**Keywords:** osteocytes, autophagy, osteogenesis, mechanical tension, FGF23

## Abstract

Mechanical stimuli control cell behaviors that are crucial for bone tissue repair. Osteocytes sense extracellular mechanical stimuli then convert them into biochemical signals to harmonize bone remodeling. However, the mechanisms underlying this process remain unclear. Autophagy, which is an evolutionarily preserved process, that occurs at a basal level when stimulated by multiple environmental stresses. We postulated that mechanical stimulation upregulates osteocyte autophagy via AMPK-associated signaling, driving osteocyte-mediated osteogenesis. Using a murine model of orthodontic tooth movement, we show that osteocyte autophagy is triggered by mechanical tension, increasing the quantity of LC3B-positive osteocytes by 4-fold in the tension side. Both *in vitro* mechanical tension as well as the chemical autophagy agonist enhanced osteocyte Fibroblast growth factor 23 (FGF23) secretion, which is an osteogenenic related cytokine, by 2-and 3-fold, respectively. Conditioned media collected from tensioned osteocytes enhanced osteoblast viability. These results indicate that mechanical tension drives autophagy-mediated FGF23 secretion from osteocytes and promotes osteogenesis. Our findings highlight a potential strategy for accelerating osteogenesis in orthodontic clinical settings.

## Introduction

Orthodontic tooth movement (OTM) is a consequence of mechanical pressure-mediated bone remodeling. There exist a tension and compression side on both sides of the root. Osteogenesis occurs on the tension side following osteoblast actuation (Tompkins, 2016). However, molecular mechanisms involved in alveolar bone remodeling during the process of OTM have not been established. In the past, osteoclasts, periodontal ligament cells and osteoblasts have been postulated to be the main cells modulating tooth movement (Xie et al., 2009; Uribe et al., 2011; McCormack et al., 2014). However, studies are evaluating the roles of osteocytes in remodeling alveolar bones during the perception of mechanical signals and regulation of OTM (Bumann and Frazier-Bowers, 2017). Osteocytes, the most common cells in bone tissues, develop from terminal differentiation of osteoblasts. These cells are interconnected by synaptic networks in the mineralized matrix of the bone interconnected into a unique anatomical network called the luminal canal system (LCS) (Bonewald, 2011; Chen et al., 2015). Consequently, studies have evaluated whether osteocytes are essential mechanosensory bone cells that can rapidly transduce mechanical signals via the LCS (Han et al., 2004). However, most research has focused on the osteocytes mechanobiology under fluid flow shear stress (FSS) (Han et al., 2004; Deepak et al., 2017; Liao et al., 2017) and few studies have investigated if osteocytes respond to other mechanical stimulation forms, such as mechanical tension (Odagaki et al., 2018; Hoshi et al., 2020). Moreover, osteocytes are vital in coordinating alveolar bone metabolism during OTM. A study investigated the role of osteocytes in osteoclastic bone resorption during orthodontic tooth movement. Genetically modified mice that specifically ablated osteocytes was used in this study. It was found that the ablation of osteocytes significantly diminished the distance of tooth movement. In addition, the number of osteoclasts in the compressed part of the alveolar bone also decreased significantly (Matsumoto et al., 2013). Previous studies of our group have demonstrated that osteocytes promote osteoclast production through autophagy-mediated secretion of RANKL under mechanical pressure (Li et al., 2020). However, the underlying mechanism by which osteocytes regulate osteoblasts in OTM is unclear.

Autophagy is a process by which cellular components, such as misfolded proteins and damaged organelles are packaged in autophagosomes and degraded by lysosomes (Rabinowitz and White, 2010). This process is a vital modulator of bone homeostasis as well as remodeling (Shapiro et al., 2014; Pierrefite-Carle et al., 2015). Impaired bone autophagy causes bone metabolism disorders. Age-associated suppression of osteocyte autophagy has been shown to lead to bone loss (Chen et al., 2014). Mice in which Atg7 (autophagy-related 7) is conditionally knocked out in osteocytes exhibit skeletal aging phenotypes, such as reduced osteoblasts, suppressed bone mass as well as decreased rate of bone formation (Onal et al., 2013). Hence, we postulated that autophagy is a crucial modulator of osteocytes function and bone tissue fate. Numerous studies have implicated autophagy in protein secretion, a process called “secretory autophagy” (Manjithaya et al., 2010; Dupont et al., 2011; Kraya et al., 2015; Wang et al., 2019). Therefore, there is a link between the extracellular microenvironment and intracellular autophagy.

Fibroblast growth factor 23 (FGF23) is a member of the fibroblast family of cytokines. Since FGF23 was found in the ventrolateral thalamic nucleus of the brain in 2000 (Yamashita et al., 2000), it has been proved that FGF23 is mostly high-expressed in bones and is mainly expressed by osteocytes (Shimada et al., 2004; Feng et al., 2006; Ichikawa et al., 2017). The secretion of FGF23 is one of the main inducement for both immature and mature osteocytes to regulate mineralization and phosphate homeostasis. Therefore, FGF23 could be seen as a useful marker of osteoblast function (Ubaidus et al., 2009). Among different molecules used to induce cell differentation during cell culture, Dexamethasone (Dex), a synthetic glucocorticoid, is known to be an important regulator of mesenchymal progenitor cell commitment to osteoblast, adipocyte and chondrocyte lineages. Indeed, it has been demonstrated, in many studies, that Dex regulates the osteogenesis of human MSCs and mineralization *in vitro* (Cheng et al., 1994; Jaiswal et al., 1997; Aubin, 1998; Walsh et al., 2001; Hardy and Cooper, 2011). It is also demonstrated that Dex has a pro-osteogenic effect on mouse MSCs(Edgar et al., 2007). However, according to previous studies, DEX treatment (1 μM) induced a significant decrease in cell viability and cell death in OB-6 cells,a murine osteoblast cell line (Zhang et al., 2018a; Fan et al., 2019). A study by Hao et al. showed that FGF23 could protect osteoblasts from DEX-induced oxidative damage and cell death (Ji et al., 2020). Osteocytes have been shown to control bone metabolism through the secretion of FGF23 (Bergwitz and Jüppner, 2010; Han et al., 2018) and through various types of osteocytic death (Moin et al., 2014). Notably, a 2012 study on the alveolar bone characteristics associated with the physiological movement of molar teeth in mice showed by histological and histochemical analysis of the alveolar bone around the roots of molar that osteocytes in the supporting alveolar bone on the bone forming side showed a strong positive immunoreactivity for FGF23. Therefore, we are interested in the role of FGF23 secreted by osteocytes in bone metabolism.

CARM1 (co-activation-related arginine methyltransferase 1) is a key component of mammalian autophagy. In conditions of adequate nutrition, CARM1 is stably expressed in the nucleus. However, low nutrition activates nuclear AMPK, which elevates CARM1 protein level and enhances histone H3 Arg17 dimethylation through *vi*a FOXO3a phosphorylation. In turn, CARM1 activates TFEB-mediated transcription of autophagy-related and lysosomal genes (Shin et al., 2016). AMPK can stimulate osteoblast differentiation and mineralization by inducing autophagy (Pantovic et al., 2013). Using RNA-seq to detect MLO-Y4 cells after fluid shearing force revealed AMPK signaling activation (Govey et al., 2015). Fluid shear force can promote autophagy in MLO-Y4 cells (Zhang et al., 2018b). Thus, we postulated that mechanical force activates osteocyte autophagy via AMPK signaling and promotes osteocyte-associated osteogenesis. Here, we show that mechanical tension induces autophagy in osteocytes during OTM. Moreover, we reveal that tension-mediated autophagy in osteocytes increases FGF23 secretion, promoting osteoblast formation.

## Methods and Materials

### Experimental Animals

This study adhered to guidelines by the animal care and use committee of Nanjing Medical University (Approval ID 1811055). Experimental murine orthodontic tooth movement was conducted as described previously (Hirt and Liton, 2017). Before bonding the nickel-titanium coil spring with a flowable repair resin (3 M ESPE) between the right maxillary first molar and the maxillary incisor, anesthesia of mice was done by intraperitoneal administration of chloral hydrate (5%; 0.1 ml/10 g). Force value was approximately 30 g each (Tompkins, 2016). The left upper jaw was unloaded and used as a contralateral control. Sample sizes were at least n = 3 to allow for statistical analysis.

### Micro-CT Analysis

After 7 days of OTM, mouse maxillary bones were collected and fixed in 4% PFA for 12 h. After removing the nickel-titanium coil spring, micro CT scan was performed at a resolution of 15.6 μm, 55 kvp voltage, and 145 μA current. Next, DataViewer was used to adjust the head position so that for all skulls, brain tissue faced down and the occipital bones faced back. CT Vox software was used for image reconstruction. CTAn software was used to make 2 shots in the coronal direction of the sagittal suture on all skulls. Volume measurement based on volume of interest (VOI) was done as described before (Hirt and Liton, 2017). In the sagittal plane, the alveolar bone between the mesial and distal buccal roots of the first molar was selected. Ten consecutive images, starting from disappearance of the distal buccal root were evaluated.

### ALP Staining

For frozen sections, samples fixation was done in 4% PFA, decalcified with EDTA (10%), embedded in Tissue-Tek, and sectioned at 5 μm. Section staining was done using a TRAP/ALP staining kit (WAKO) following manufacturer instructions. We analyzed alveolar bone around the tensioned side of maxillary first molar mesiobuccal root was microscopically (Olympus Optical, Japan) analyzed. The unit area of positive staining in each section was calculated. Fixing of cultured cells was done in PFA (4%) for 10 min 7 days after osteogenic induction and stained with 1-Step™ NBT/BCIP substrate solution (Thermo Scientific). Images were taken randomly, and ALP positive area of each group calculated on ImageJ.

### Immunofluorescence Staining

Immunostaining was performed following a standard protocol. The protocol used to stain sections and cells is practically very similar. Fixation of samples was done in PFA (4%) for 10 min, permeabilized using TritonX-100 (0.5%) for 10 min, blocked with goat serum (BOSTER, AR0009) for 30 min at room temperature, and incubated in the presence of primary antibodies against LC3B at 4°C overnight. After washing thrice with PBS, they were incubated in the presence of a Cy3-labeled secondary antibody (Proteintech, SA00009-2) at 37°C for 1 h. They were then rinsed thrice and counter-stained with DAPI (VECTASHIELD, H-1500) for 30 s. They were then examined by laser confocal microscopy (OLYMPUS, FV3000) and the number of LC3B-positive osteocytes determined.

### Cell Culture

MLO-Y4, a murine osteocyte cell line was grown on mouse tail collagen type I (BD Biosciences, Bedford, MA) coated dishes and maintained in α-minimum essential medium (α-MEM, Gibco) supplemented with L-glutamine, nucleosides, 5% FBS (Sciencell), calf serum (5%; CS, Gibco), and 1% penicillin/streptomycin (Hyclone). The pre-osteoblast cell line MC3T3-E1 was purchased from the Chinese Academy of Sciences. Pre-osteoblast isolation from skull bones of newborn mice was as follows: eight newborn mice (purchased by the Experimental Animal Center of Nanjing Medical University) were sacrificed and disinfected in 75% alcohol 5–10 min. The top skin was incised to take out the skull, then remove the periosteum and surrounding connective tissue. The cartilage between the sutures was removed as well.Next, soak and rinse the bones several times with sterile PBS until there is no blood on the bones, then move to another sterile plate. Ophthalmic scissors were then used to cut the bone block into 1 mm^3^ pieces or smaller. 0.25% trypsin+0.02% EDTA compound enzyme digestion solution was then added for digestion for 10 min at 37°C. Sloughed cells were pipetted to enhance digestion, and the digestion solution containing cells carefully transferred into a centrifuge tube. Supernatants were discarded while the pellet was evenly resuspended in complete culture media before seeding in tissue culture dishes. Cells were incubated in a humidified 5% CO_2_ atmosphere at 37°C. Experiments were repeated 3 times.

### Dynamic Loading and CM Collection

MLO-Y4 osteocyte-like cells were allocated into a control group (no loading to cultured cells/0 h) and four treatment groups and seeded on 6-well flexible bottom plates at 1 × 10^4^ cells/well. They were then cultured overnight to near-confluence and then serum-starved for 8 h before being tensioned. A Flexcell strain unit (Flexcell FX-5000T; Flexcell Corp., Burlington, NC, United States) was used to cyclically stretch the treatment group for 15, 30, 60, and 120 min in the cell culture medium at a frequency of 1Hz. Culture of the control group cells was done on similar plates and kept in the same incubator without cyclic stretching. Three biological replicates were cultivated and measured for each analytical method. After exposure to mechanical tensile stress, The MLO-Y4 cells were seeded in fresh α-MEM medium for 12 h. Conditioned media was then collected from each group and stored in a refrigerator at -80°C for use within 1 week.

### ALP Activity Assay

MC3T3-E1 cells and murine primary osteoblast precursor cells were harvested using trypsin and ethylenediaminetetraacetic acid. They were then lysed in 1% Triton for 30 min. Then the ALP activity was measured by a colorimetric assay of enzyme activity using a commercially available assay kit (Nanjing Jiancheng Bioengineering Institute, China, A059-2). 5 μL of cell lysate was then mixed with 50 μL of the buffer, followed by incubation in a 96-well plate at 37°C for 15 min without light. 150µL of spectrophotometric developer was then added and ALP-associated absorbance read on a plate reader at 520 nm. ALP activity (U/gprot) was quantified with the equation:

### Enzyme-Linked Immunosorbent Assay

ELISA was used to quantify FGF23 secretion by MLO-Y4 cells. Cells were inoculated on 6-well collagen type I-coated BioFlex culture plates and incubated as described above. Culture media was then collected, centrifuged, and the supernatant subjected to FGF23 quantitative ELISA using elabscience Mouse FGF23(Fibroblast Growth Factor 23) ELISA Kit immediately. Experimental procedures were as described by the manufacturer.

### Quantitative PCR

Cells were harvested and total RNA extracted at the indicated timepoints using Takara Minibest Universal RNA extraction kit (Catalog number: 9767), then transformed to cDNA using Takara Primescript RT Master Mix kit (Takara, RR036A). The mRNA levels were then measured on an ABI (QuantStudio 7) RT-PCR system using SYBR-Green (Roche) and following manufacturer instructions. Primer sequences are shown on Table 1.

**TABLE 1:** The primer sequences used in this study are listed as follows.

Genes	Forward primer (5′–3′ )	Reverse primer (5′–3′ )
ATG4	GCT​GGT​ATG​GAT​TCT​GGG​GAA	TGG​GTT​GTT​CTT​TTT​GTC​TCT​CC
ATG5	TGT​GCT​TCG​AGA​TGT​GTG​GTT	GTC​AAA​TAG​CTG​ACT​CTT​GGC​AA
ATG7	GTT​CGC​CCC​CTT​TAA​TAG​TGC	TGA​ACT​CCA​ACG​TCA​AGC​GG
LC3B	TAA​TCT​GAG​CAA​TGC​GAT​TGT​GG	AGA​TGG​ACG​GAG​TAT​AGC​GAA​AA
P62	AGG​ATG​GGG​ACT​TGG​TTG​C	TCA​CAG​ATC​ACA​TTG​GGG​TGC
ULK1	AAG​TTC​GAG​TTC​TCT​CGC​AAG	CGA​TGT​TTT​CGT​GCT​TTA​GTT​CC
RUNX2	TTA​CCT​ACA​CCC​CGC​CAG​TC	TGC​TGG​TCT​GGA​AGG​GTC​C
OCN	CTG​ACC​TCA​CAG​ATG​CCA​AGC	TGG​TCT​GAT​AGC​TCG​TCA​CAA​G
OPN	AGC​AAG​AAA​CTC​TTC​CAA​GCA​A	GTG​AGA​TTC​GTC​AGA​TTC​ATC​CG
ALP	TCC​TGA​CCA​AAA​ACC​TCA​AAG​G	TGC​TTC​ATG​CAG​AGC​CTG​C
FGF23	GAC​CAG​CTA​TCA​CCT​ACA​GAT​C	GTA​ATC​ATC​AGG​GCA​CTG​TAG​A
GAPDH	AGG​TCG​GTG​TGA​ACG​GAT​TTG	TGT​AGA​CCA​TGT​AGT​TGA​GGT​CA

### Western Blotting Analysis

Cells were harvested, washed, and lysed using whole cell lysis assay kit (KeyGEN BioTECH, China, KGP250). Protein concentration was assessed using a Bradford protein analysis kit (Beyotime, China, P0006). Sixty micrograms of protein were loaded into each well and resolved by 12% SDS-PAGE before transfer onto PVDF membranes (Millipore, Billerica, MA). Membrane blocking was done for 2 h using 5% skim milk followed by overnight incubation in the presence of various primary antibodies at 4°C. Details of the primary antibodies used are listed on Table 2. They were then washed with 0.1% TBST and incubated in the presence of a suitable secondary antibody for 1 h at room temperature. Signal development was done using a Tanon High-sig ECL Western Blotting Substrate (180–501) followed by imaging on a Tanon 5200 chemiluminescence imaging system. GAPDH was the loading control.

**TABLE 2:** The antibodies used in this study are listed as follows.

Antibodies	Source	Identifier	Dilution concentration
anti-LC3B	Abcam	Cat.# ab51520	1/2000 for WB; 1/1000 for IF
anti-ATG7	Abcam	Cat.# ab133528	1/1000
anti-AMPKα	CST	Cat.# 5831	1/1000
anti-P62	CST	Cat.# 5114	1/1000
anti-FGF23	Abcam	Cat.# ab98000	1/1000
anti-p-AMPKα	CST	Cat.# 2535	1/1000
anti-CARM1	Proteintech	Cat.# 55246-1-AP	1/1000
anti-GAPDH	Beyotime	Cat.# AG019	1/1000

### Statistical Analysis

Data are shown as either mean ± s.e.m. or mean ± s.d for a minimum of three independent experiments, unless otherwise stated. Comparisons of means between and among groups was done by the student’s t-test or one-way ANOVA, respectively. *P=*<0.05 was considered significant.

## Results

### Osteocyte Autophagy Is Activated During Orthodontic Tooth Movement

**FIGURE 1:**
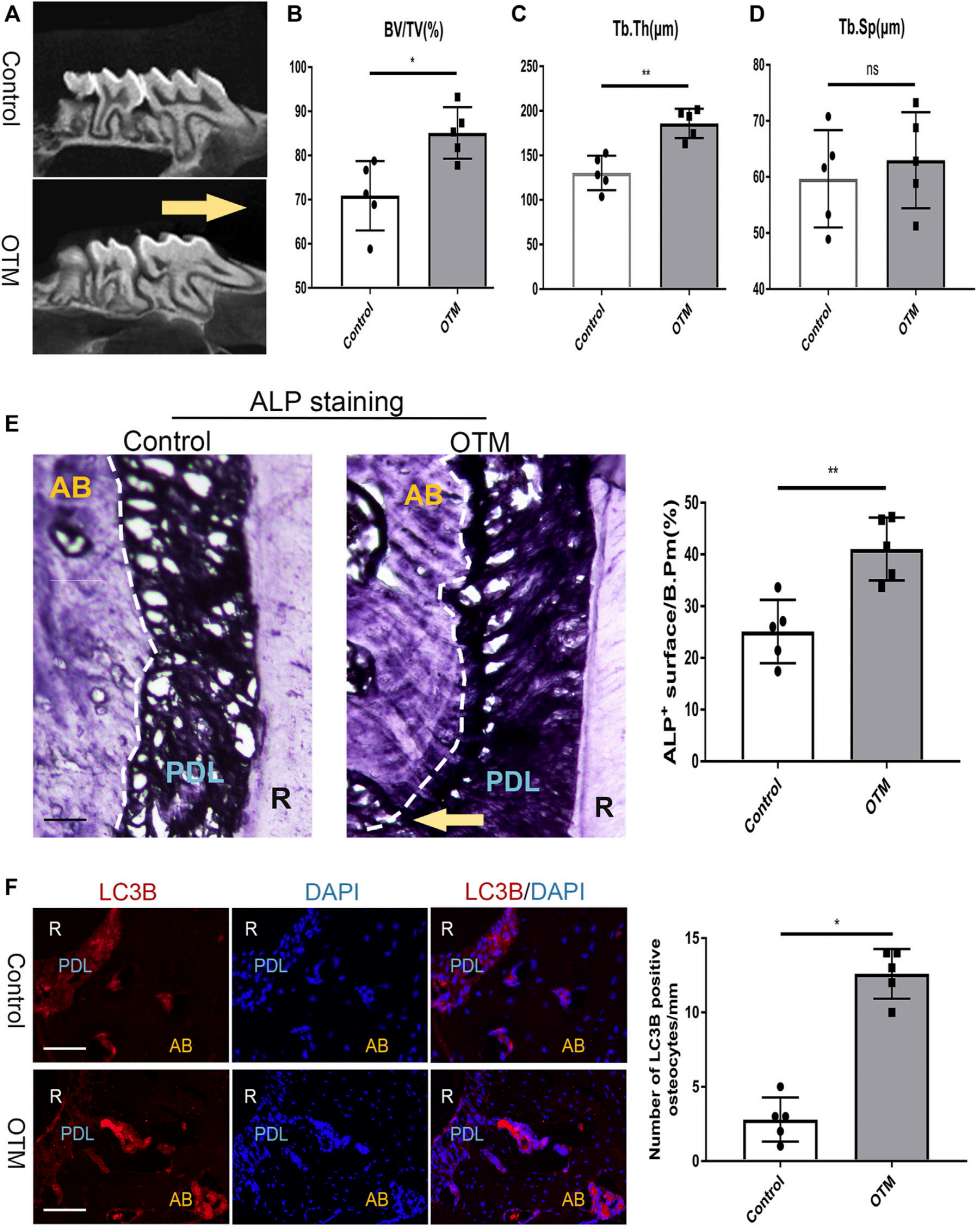
Orthodontic tooth movement activated osteocyte authophagy. **(A)** Representataive images showing the distance between the first and second maxillary molars after 7 days of orthodontic tooth movement. Yellow arrow showed the direction of tooth movement. Scale bar = 500 μm, *n* =5. **(B–D)** Micro-CT analysis of avveolar bone volume. Ration of trabecular bone volume/total volume(B-V/TV%), trabecular thickness(Tb.Th) and trabecular separation (Tb.Sp) were measured.**(E)** Frozen sections of the maxillary first molar were stained by ALP. The quantification of ALP + surface/B.Pm(%). Yellow arrow showed the direction of tooth movement. Scale bar = 50 μm. **(F)** maxillary bone adjacent frozen sections were subjected to IF staining of osteocytes with anti-LC3B. DAPI was counterstained to show the nuclei (blue). The quantification of LC3B + osteocytes/mm. White dashed lines respresented the bone surface. Scale bar = 50 μm. All data were showed as mean ± SD.*p ˂ 0.05 *vs*. control.

To study if orthodontic tooth movement process involves osteocyte autophagy, a previously reported OTM mouse model was used (Li et al., 2020). Seven days later, the first upper right molar was moved mesially (Figure 1A) and bone volume around root furcation determined by Micro-CT. It was found that Bone Volume (BV)/Total Volume (TV)% as well as Trabecular Thickness (Tb⋅Th) were markedly increased, although Trabecular separation (Tb.Sp) differences were not statistically significant (Figures 1B–D). To assess osteoblasts formation and changes in osteocyte autophagy, maxillary bone adjacent frozen sections were cut. ALP staining showed that the area of the alveolar bone on the tension side of the distobuccal root of the maxillary first molar increased significantly (Figure 1E). Immunofluorescence (IF) showed a high number of LC3B-positive osteocytes, which were near these osteoblasts (Figure 1F), implying enhanced autophagy in bone cells during the OTM process. Therefore, OTM increases autophagic levels in osteocytes.

### *In vitro* Mechanical Tension Induces Autophagy in Osteocytes

**FIGURE 2:**
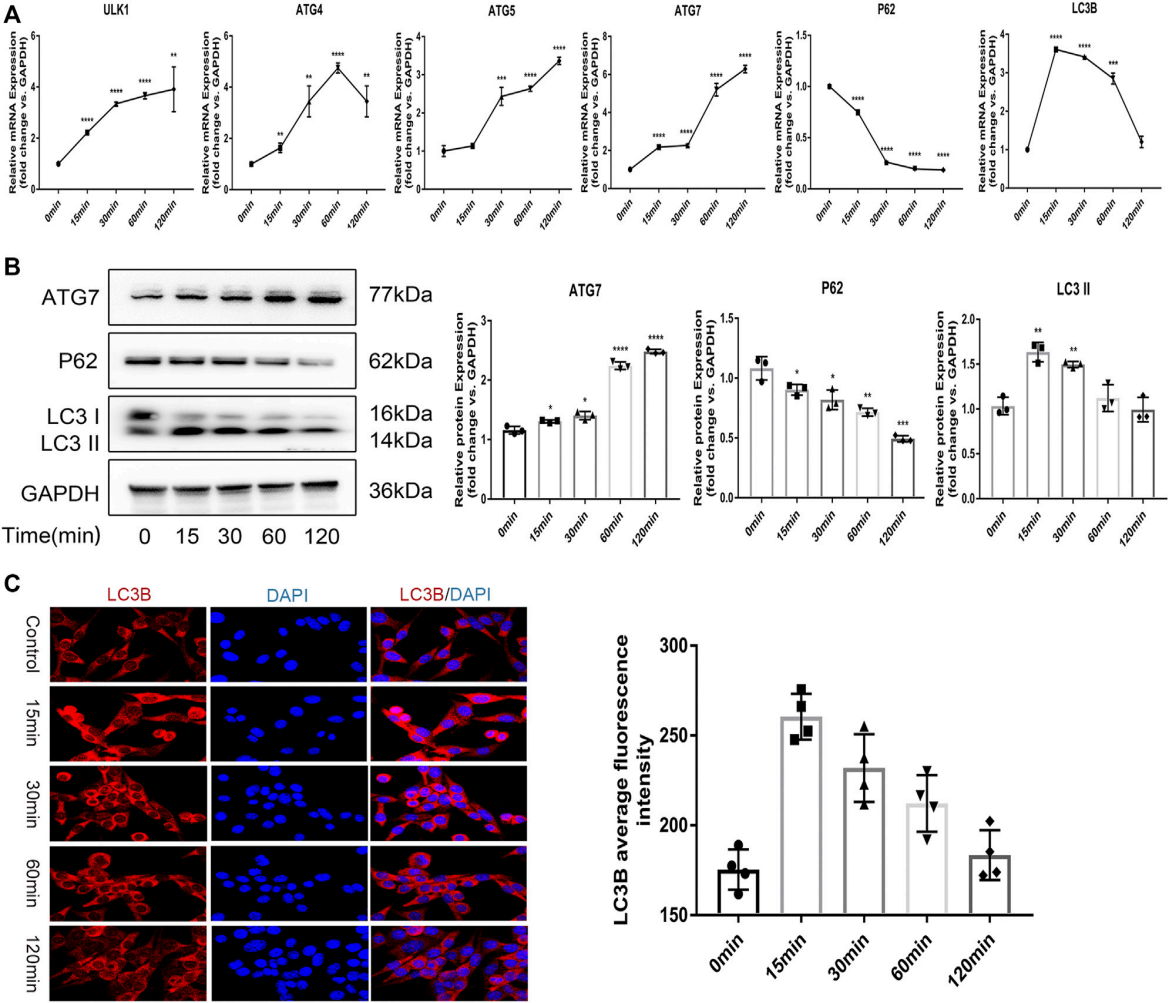
In vitro mechanical tension induces autophagy in osteocytes **(A)** The mRNA expression of ATG4 ATG5 ATG7 LC3B ULK1 were detected by realtime RT-PCR. GAPDH was used for normalization. **(B)** The protein expression of ATG7 P62 and LC3B were measured by western blot. The quantification of ATG7 P62 and LC3BII. GAPDH was used for normalization. **(C)** Immunofluorescence staining was used to visualize autophagosomes (respresented by LC3 positive spots). Scale bar = 10 μm. All cell experiments were repeated 3 times and each time they were performed in at least 3 replicate wells. All data were showed as mean ± SD. *p ˂ 0.05 *vs*. control.

To determine if mechanical tension induces osteocyte autophagy *in vitro*, we used a Flexcell^®^ FX tensile stress loading system to establish a tensile stress loading model using MLO-Y4, a bone cell line and evaluated mRNA as well as protein levels of various autophagy markers. Tension significantly elevated mRNA expression levels of ATG5, ATG4, ATG7, LC3B as well as ULK1 (Figure 2A). Western blot analysis confirmed elevated levels of the autophagy markers ATG7 and LC3BII, while the autophagy substrate, P62 gradually decreased (Figure 2B). P62 is also called SQSTM1. It is expressed in a variety of cells and tissues and it can participate in a variety of signal transduction processes. P62 can connect LC3 and the ubiquitinated substrate, P62 and its bound polyubiquitinated proteins are integrated into autophagosomes and are degraded in autophagic lysosomes, so P62 is the substrate of autophagy.When autophagy is activated, autophagosomes fuse with lysosomes, proteins such as P62 or organelles in autophagic vesicles are degraded by lysosomes, and P62 level decreases. When autophagy is inhibited, autophagosomes accumulate and P62 level increases. Therefore, P62 can be used as an indicator of autophagy ability, and the reduction of P62 expression by Western blotting technique can reflect the degree of autophagy activity. To assess autophagic flux, we futher used IF to visualize autophagosomes (indicated by LC3-positive puncta) and observed significant increase in autophagosomes after tension, beginning after 15 min and peaking at 60 min before gradual decrease, indicating that mechanical tension induces osteocytic autophagy in osteocytic-like MLO-Y4 cells *in vitro*. In addition, The magnification in the IF image analysis is ×400.

### Mechanical Tension Promotes Osteocyte-Mediated Osteogenesis

**FIGURE 3:**
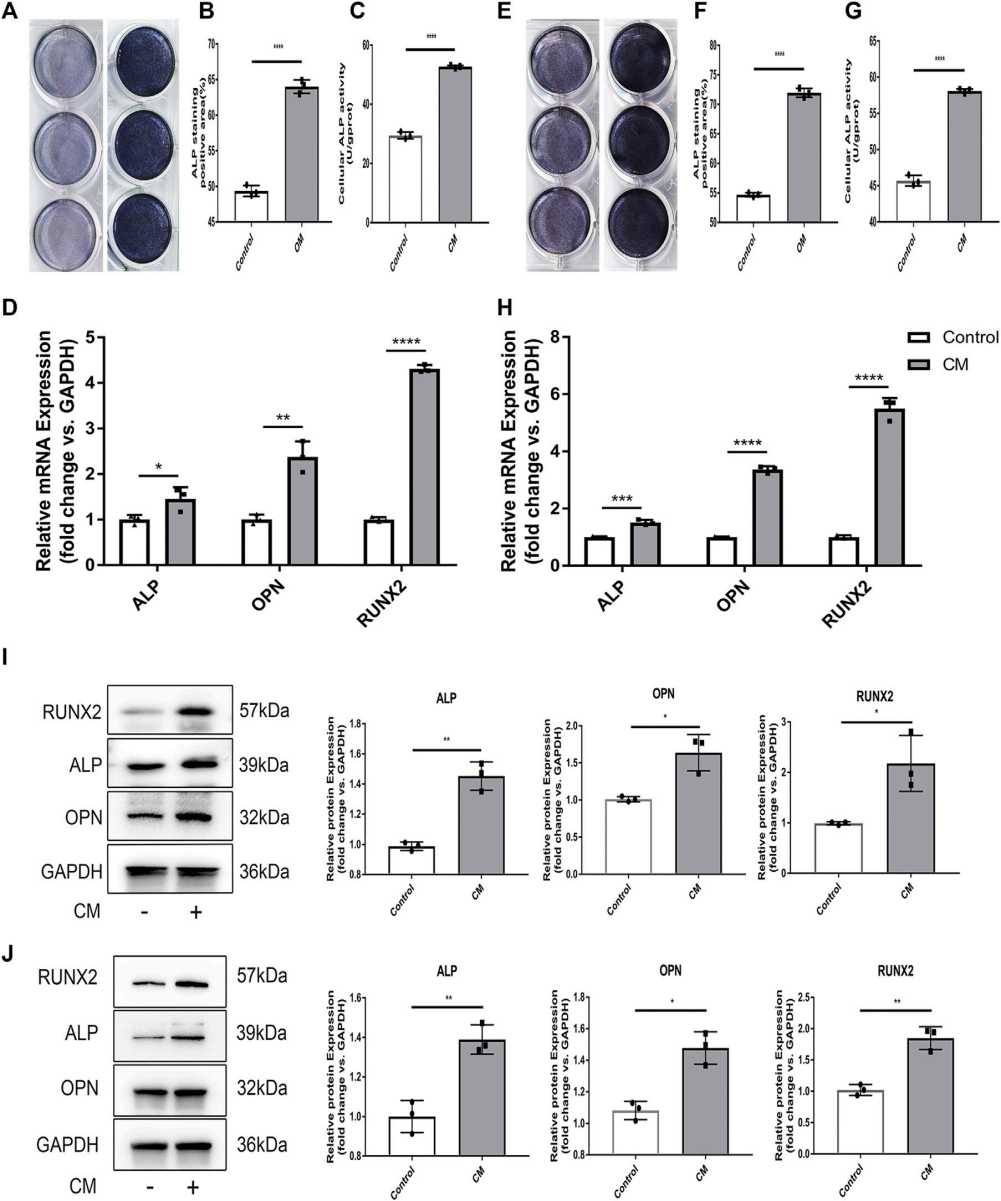
Mechanical tension promotes osteocyte-mediated osteogenesis. **(A)** ALP staining of 3T3-E1 after induction of differenation with CM from MLO-Y4. **(B)** The quantification of ALP+ surface/B.Pm(%) of 3T3-EI after induction of differentiation with CM from MLO-Y4. **(C)** Cellular ALP activity (U/gprot) of 3T3-E1 after induction of differentiation with CMfrom MLO-Y4. **(D)** The expression level of osteoblastic markers as ALP OPN RUNX2 of 3T3-E1 was detected by RT-PCR. GAPDH was used for normalization. **(E)** ALP staining of primary osteoblast precursor cells after induction of differenation with conditioned medium (CM) from MLO-Y4. **(F)** The quantification of ALP + sueface/B.Pm(%) of primary osteoblast precursor cells after induction of differentiation with CM from MLO-Y4. **(G)** Cellular ALP activity (U/gprot) of primary osteoblast precursor cells after induction of differentiation with CM from MLO-Y4. **(H)** The expression level of osteoclastic markers of as ALP OPN RUNX2 of primary osteoblast precursor cells was detected by RT-PCR. GAPDH was used for normalization. **(I)** The protein expression of ALP OPN and RUNX2 of 3T3-E1 were measured by western blot. The quantification of ALP OPN and RUNX2. GAPDH was used for normalizatio. **(J)** The protein exoression of ALP OPN and RUNX2 of primary osteoblast precursor cell were measured by western blot. The quantification of ALP OPN and RUNX2. GAPDH was used for normalization. All cell experiment were repeated 3 times and each time they were performed at least 3.

Given that osteocytes influence osteogenesis (Bays et al., 2017), we examined if tension affects the ability of osteocytes to regulate bone remodeling. To this end, MC3T3-E1, an osteoblast precursor cell line was seeded in conditioned medium (CM) derived from MLO-Y4 cells exposed to tension and subjected them to alkaline phosphatase (ALP) staining. ImageJ analysis revealed that relative to the control group, the CM group had stronger ALP levels, indicating that the osteocyte conditioned media from MLO-Y4 cells subjected to tension significantly enhanced early osteogenesis in MC3T3-E1 osteoblasts. Analysis of ALP activity in the 2 groups revealed significantly higher ALP activity in CM group relative to the control group, which is consistent with RT-qPCR results of the expression of the osteoblast markers ALP, OPN and RUNX2. Consistently, western blot analysis revealed significantly elevated levels of the osteogenic related proteins, ALP and RUNX2 (Figure 3E). To verify these findings, we cultivated murine primary osteoblast precursor cells in CM. ALP staining as well as ALP activity assays revealed that osteoblast precursor cells cultured in CM had enhanced osteogenesis ability (Figures 3F–I), which is consistent with RT-qPCR and western blot results (Figure 3J).

### Autophagy Up-Regulated Osteocyte-Associated Osteogenesis

**FIGURE 4:**
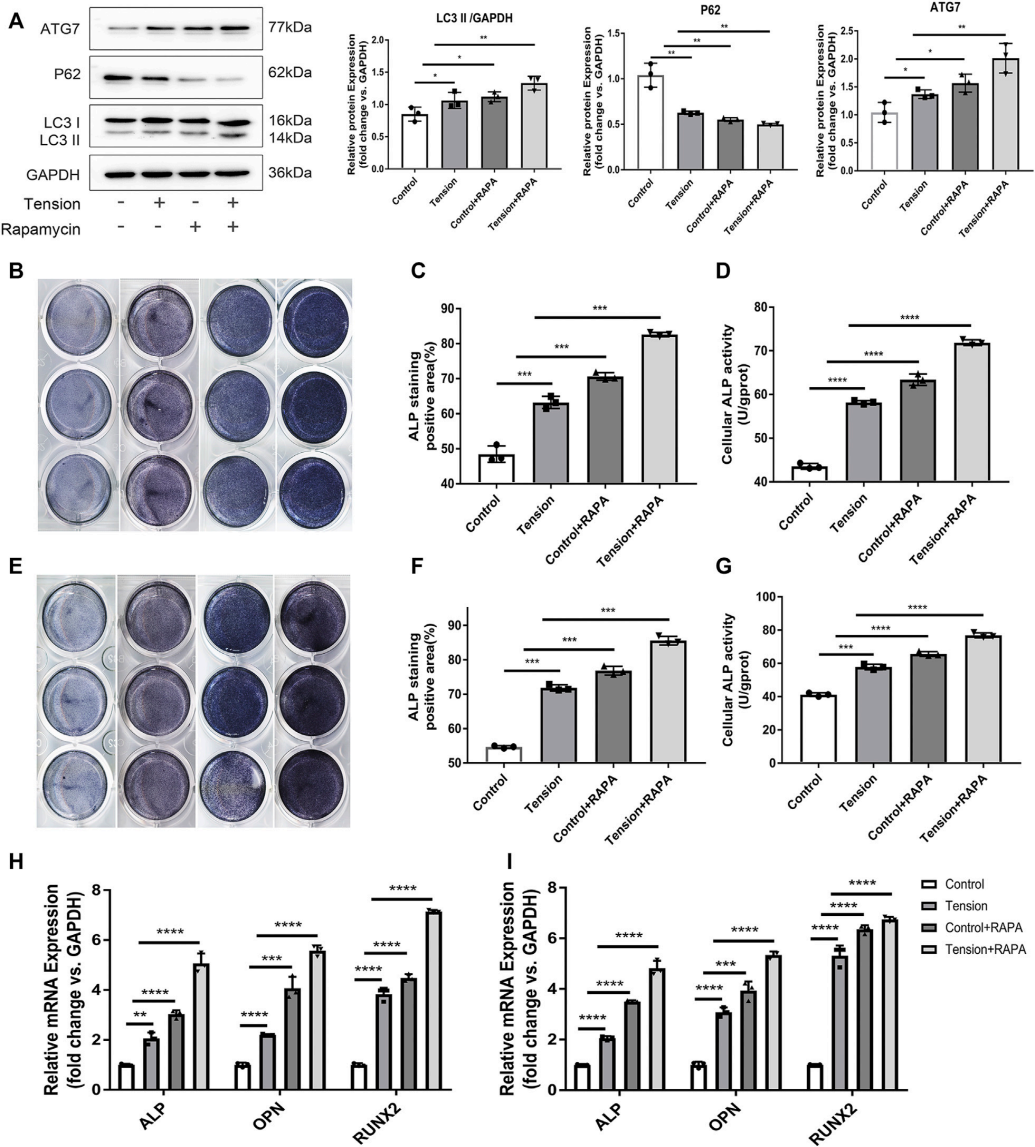
Autophagy up regulated osteocyte-associated osteogenesis. **(A)** The cells were incubated with Rapamycin for 6 h before subjected to mechanical tension. LC3B ATG7 and P62 were detected by western Blot. GAPDH was used for normalization. **(B)** ALP staining of 3T3-E1 after induction of differentiation with CM from MLO-Y4 in the indicated groups. **(C)** The quantification of ALP + surface/B.Pm(%) of 3T3-E1 after induction of differentiation with CM from MLO-Y4 in the indicated groups. **(D)** Cellular ALP activity (U/gprot) of 3T3-E1 after induction of differentiation with CM from MLO-Y4 in the indicated groups. **(E)** ALP staining of primary osteoblast precursor cells after indiction of differentiation with CM from MLO-Y4 in the indicated groups.**(F)** The quantification of ALP + surface/B.Pm(%) of primary osteoblast precursor cells after induction of differentiation with CM from MLO-Y4 in indicated groups. **(H)** The expression level of osteoblastic markers of 3T3-E1 was detected by RT-PCR. GAPDH was used for normalization. **(I)** The expression level of osteoblastic markers of primary osteoblast precursor cells was detected by RT-PCR GAPDH was used for normalization. All cell experiments were repeated 3 times and each time they were performed in at least 3 replicate wells. All data were showed as mean ± SD. *p ˂ 0.05 *vs*. control.

To investigate if autophagy is involved in osteocyte-associated osteogenesis, the MLO-Y4 cells were treated with rapamycin to activate autophagy before the application of tension. Western blot analysis revealed that rapamycin enhances mechanically-initiated autophagy (Figure 4A). ALP staining revealed that relative to controls, MC3T3-E1 cells cultivated in CM media from tension-exposed MLO-Y4 (Figures 4B–D), enhances the osteogenic function of osteoblasts. Results from similar experiments using murine osteoblast precursor cells yielded identical results (Figures 4E–G). Indicating that both mechanical and chemical induction of autophagy promotes osteoblasts development.

### Mechanical Tension-Iniated Autophagy Promotes FGF23 Secretion

**FIGURE 5:**
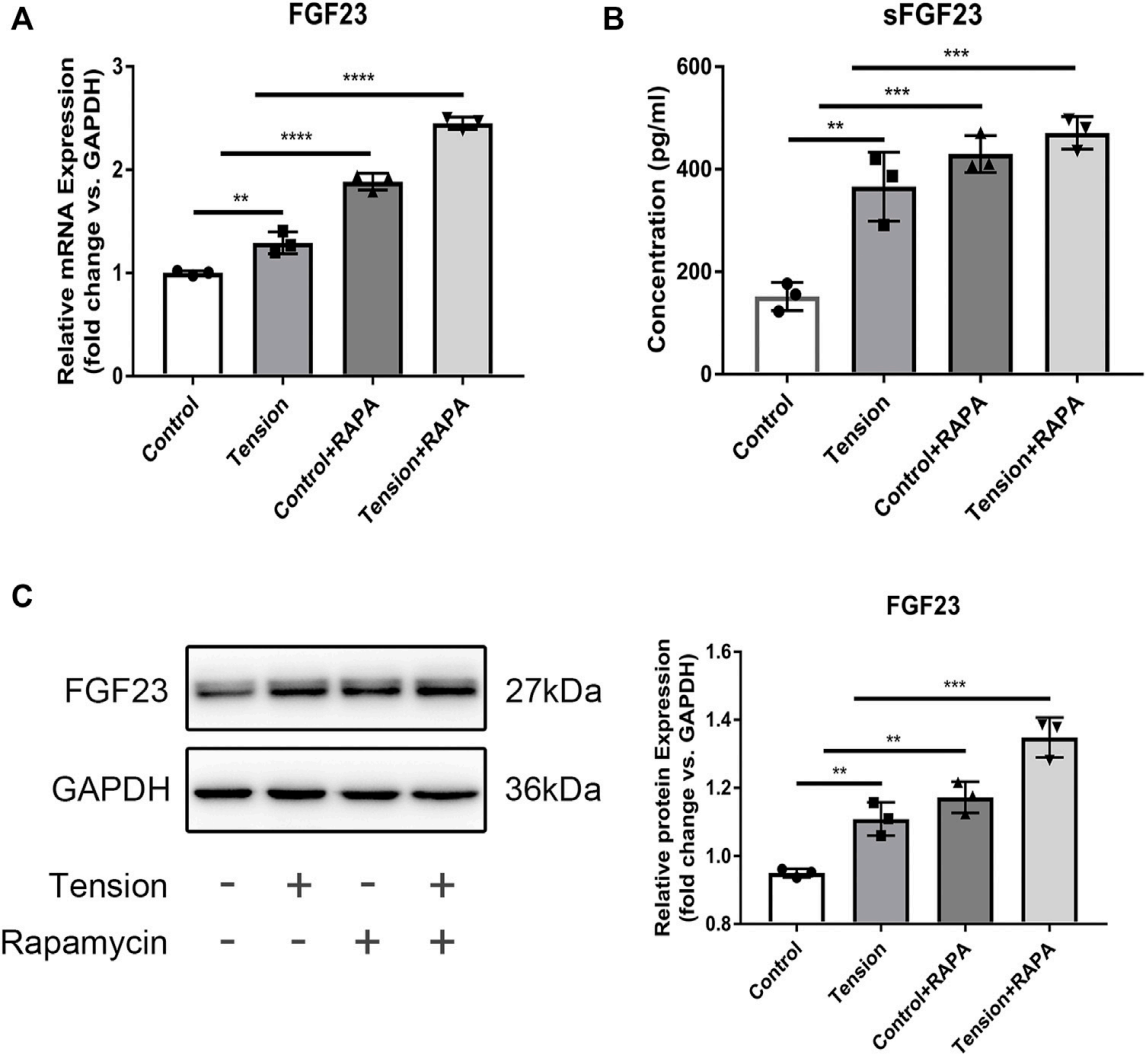
Mechanical tension-iniated autophagy promotes FGF23 secretion. **(A)** The mRNA expression level of FGF23 MLO-Y4 after mechanical tension was measured by RT-PCR. GAPDH was used for normalization. **(B)** The expression of soluble FGF23 in the supernatant of MLO-Y4 after mechanical tension was detected by ELISA. **(C)** Western blot analysis of total protein expression of FGF23. GAPDH was used for normalization. All cell experiments were repeated 3 time and each time they were performed in at least 3 replicate wells. All data were showed as means ± SD. *p ˂ 0.05 *vs*. control.

Autophagy has been implicated in secretion of proteins (Manjithaya et al., 2010; Dupont et al., 2011; Kraya et al., 2015; Wang et al., 2019). FGF23 is recognized as an important regulator of phosphate and calcium homeostasis and is mainly secreted by osteocytes (Feng et al., 2006; Edmonston and Wolf, 2020)Therefore, to determine if autophagy affects osteoblast function via protein secretion, we evaluated FGF23 expression in MLO-Y4 cells. RT-qPCR analysis showed that tensile stress increases FGF23 mRNA levels, which is further enhanced by rapamycin (Figure 5A). Similar observations were made using ELISA (Figure 5B). In addition, Western Blot results showed that the protein expression of FGF23 increased when tension or rapamycin was added (Figure 5C).

### Mechanical Tension Triggers MLO-Y4 Autophagy via AMPK Signaling

**FIGURE 6:**
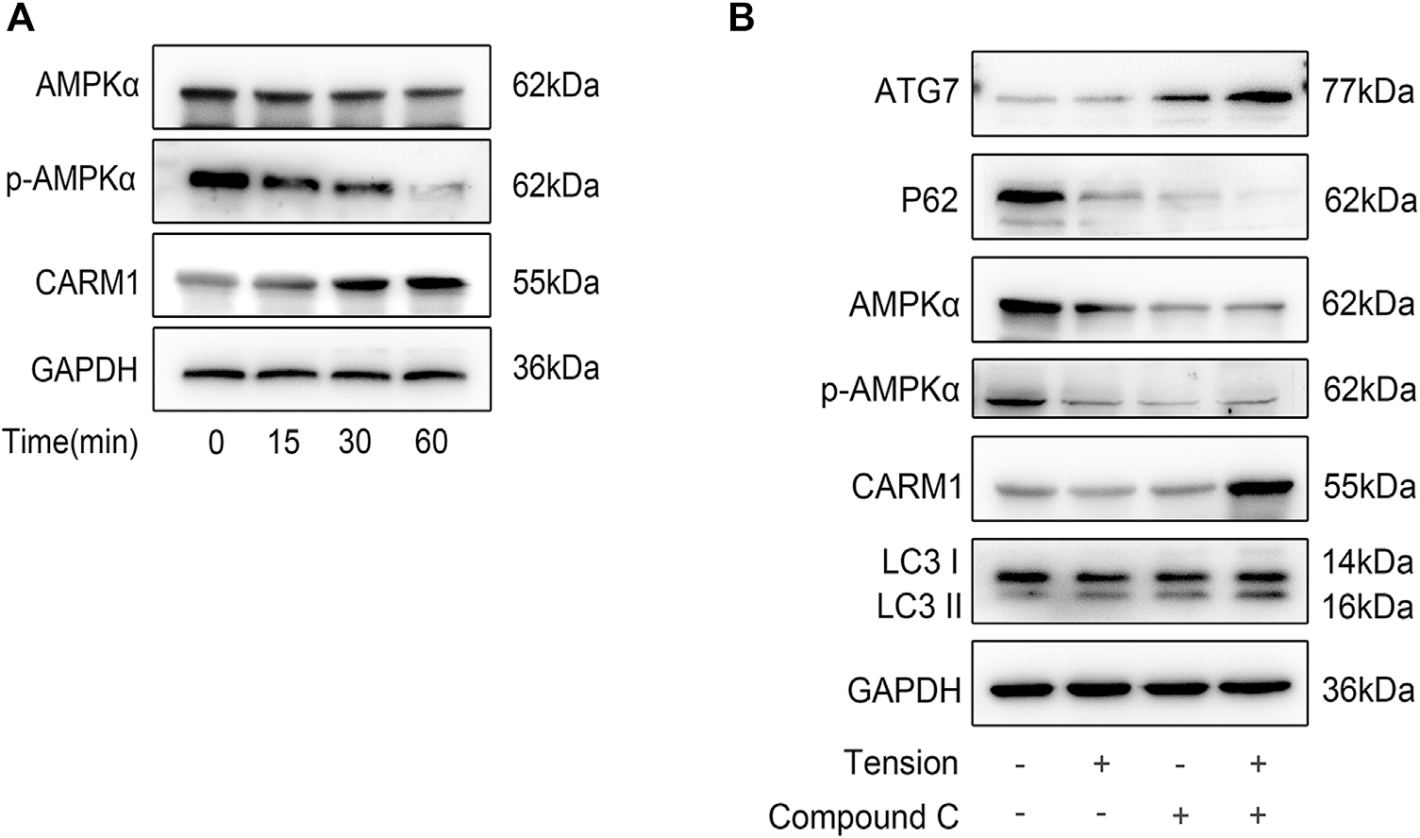
Mechanical tension tiggers MLO-Y4 autophagy *via* AMPK signaling. **(A)** The expression of the signaling molecules as AMPLα, p-AMPKα, CARM1 were detected by western blot. **(B)** Western blot analysis of AMPLα, p-AMPKα, CARM1 ATG7, P62, and LC3B in MLO-Y4 after the treatment of tension, compound C or both. All cell experiments were repeated 3 time and each time they were performed in at least 3 replicated wells.

AMPK signaling is reported to promote autophagy, which is a key modulator of metabolism and energy homeostasis (Shin et al., 2016). Because mechanical stimulation activates AMPK, which stimulates actomyosin contractility, glucose uptake, and ATP production, and that the increased energy strengthens adhesion complexes and actin cytoskeleton (Zhao et al., 2002), we evaluated if AMPK is involved in tension-mediated autophagy. Mechanical tension suppressed AMPK protein expression as well as phosphorylation time-dependently, but increased CARM1 expression (Figure 6A). Next, we used dorsomorphin (compound C) to inhibit AMPK in MLO-Y4 cells before applying tension and observed reduced AMPK expression and phosphorylation, while ATG7 and LC3BII expression levels were increased (Figure 6B). It should be noted that in the experimental results shown in this part, the tension “+” marked in the second and fourth columns in Figure 6B refers to the results obtained by collecting the cell sample after the tension is loaded for 60 min. Together, these results indicate that mechanical tension increases bone cell autophagy via AMPK signaling involving AMPK and CARM1.

## Discussion

Osteocytes have an important role in the function of bone metabolism. Osteocytes, which account for 95% of all bone cells, also regulate orthodontic tooth movement (Bergwitz and Jüppner, 2010; Moin et al., 2014) and can sense and transduce mechanical signals to coordinate bone remodeling (Bonewald, 2006). There are still few studies investigating the mechanobiology of osteocytes under mechanical tension. Here, we found that the osteocytic-like, MLO-Y4 cells can sense mechanical tension. A recent study showed that MLO-Y4 cell-secreted exosomes exposed to mechanical tension contribute to HPDLSC proliferation as well as osteogenic differentiation (Lv et al., 2020). In this study, we showed that mechanical tension increases osteocyte autophagy via AMPK signaling involving AMPK and CARM1. (Shin et al., 2016)(Pantovic et al., 2013)Our findings may expand the study of bone cell biology under mechanical tension, which is crucial in the field of orthodontics. It is reported that mechanical stimulation induces the autophagic process in various cells, including renal epithelial cells (Orhon et al., 2016), trabecular meshwork cells (Hirt and Liton, 2017), as well as vascular endothelial cells (Vion et al., 2017). Therefore, in spite of various mechanical stresses, autophagy, an evolutionarily preserved process, is a form of cell response to the external physical environment. Therefore, it is important to study the occurrence as well as role of mechanical stress-initiated autophagy in bone remodeling. We found that mechanical tension markedly elevates bone cell autophagy *in vivo* and *in vitro,* which is consistent with a previous study by Zhang (Zhang et al., 2018b). These findings imply that flow shear stress induces protective autophagy in osteocytes. However, bone metabolism during OTM is associated with multiple cells, and the likelihood that autophagy regulates alveolar remodeling in other cells cannot be ruled out. Indeed, we observed many LC3B-positive periodontal ligament cells. To verify the importance of osteocyte autophagy in a mechanical environment, future studies can use the DMP1-Cre; ATG7 flox/flox mouse for orthodontic tooth movement. Although osteoblast and bone density reduction have been reported (Onal et al., 2013; Piemontese et al., 2015), consistent with our postulate, it has not been determined whether orthodontic tooth movement speed is affected. Because FGF23 can regulate the function of osteoblasts (Ji et al., 2020), we measured its secretion in MLO-Y4 cell culture medium and observed its upregulation, which is consistent with past findings (Cheung et al., 2021). Therefore, we hypothesized that tension would increase the secretion of FGF23 by osteocytes, thereby promoting osteoblast development. Previous studies have shown that high serum FGF23 levels in humans can lead to hypophosphatasia rickets (Guo and Yuan, 2015). In contrast, some *in vitro* experiments have shown that FGF23 promotes osteoblast proliferation (Shalhoub et al., 2011). Thus, we speculate that this may be related to the concentration of FGF23 and through which pathway that it take effects. At low concentrations, increased local secretion of FGF23 by osteocytes may promote osteoblast differentiation and bone formation by acting diffusely on neighbouring cells, whereas at a high concentration, systemic overproduction of FGF23 enters the circulation and acts on target organs such as the kidney, inhibiting renal phosphorus reabsorption and leading to hypophosphatasia rickets. However, The exact machanism still wait to be proven by further studies. Autophagy is implicated in protein secretion as well as transport in various cells. Autophagy has been shown to regulate Acb1 secretion in starved *Dictyostelium discoideum* cells (Manjithaya et al., 2010). In endothelial cells, autophagy-dependent secretory granules extracellularly secrete VWF, promoting blood vessel walls healing (Torisu et al., 2013). In tumor cells, autophagy releases cargo into the extracellular space through the autophagy-MVB-exosomal pathway (Wang et al., 2019).

Interactions between autophagy and mechanical tension is vital for regulating the secretory capacity of osteocytes. We reveal that both tension- and chemically-induced autophagy enhances FGF23 secretion in bone cells and promotes osteoblast formation. Due to the important function of osteoblasts in regulating OTM occurrence, we postulate that autophagy in osteocytes may be a potential target for OTM regulation, a process that is similar to that of cancer treatments that are in clinical trials (Niu et al., 2019). In future research, we will search for more easily accessible interventions to regulate osteocyte autophagy to regulate alveolar bone metabolism to speed up OTM.

## Data Availability

The original contributions presented in the study are included in the article/Supplementary Material, further inquiries can be directed to the corresponding author.

## Abbreviations

Atg7, autophagy-related 7; ALP, alkaline phosphatase; BV, Bone Volume; CARM1, co-activation-related arginine methyltransferase 1; CM, conditioned medium; DEX, dexamethasone; ELISA, Enzyme-linked immunosorbent assay; FGF23, Fibroblast growth factor 23; FSS, fluid flow shear stress; IF, Immunofluorescence; LCS, lacunal canaliculus system; OTM, Orthodontic tooth movement; qPCR, Quantitative PCR; Tb⋅Th, Trabecular Thickness; Tb.Sp, Trabecular separation; TV, Total Volume.
