# Supplementary material for: Osteocytes Enhance Osteogenesis by Autophagy-Mediated FGF23 Secretion Under Mechanical Tension
Source: Front Cell Dev Biol. 2022 Jan 31;9:782736. doi: 10.3389/fcell.2021.782736 (PMC8841855; doi:10.3389/fcell.2021.782736)
Supplement: Supplementary file 1 [file DataSheet2.PDF]

S2 Preliminary experiments on the mRNA results of the housekeeping gene (GAPDH) in different cell lines in different treatments

A

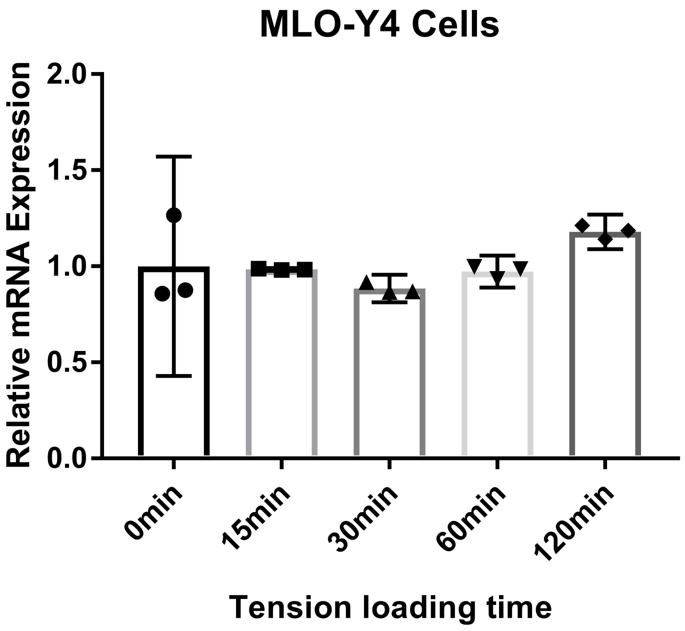

B

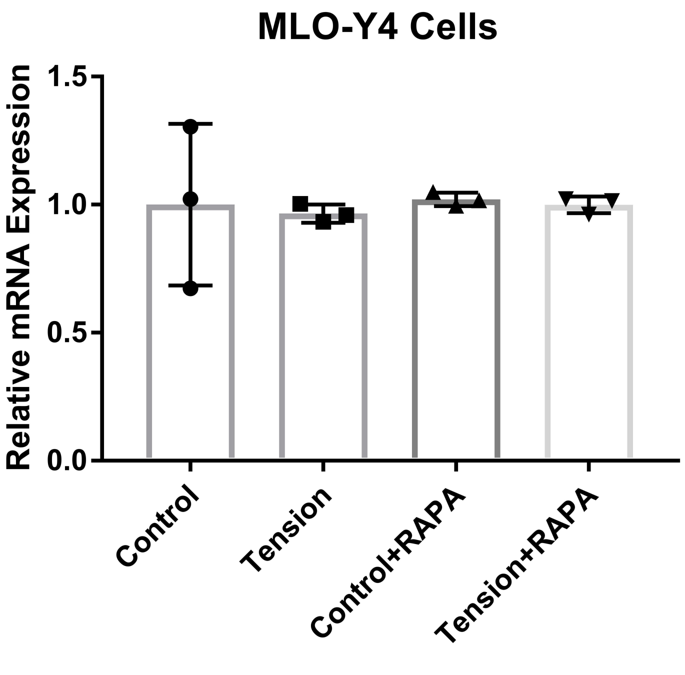

C

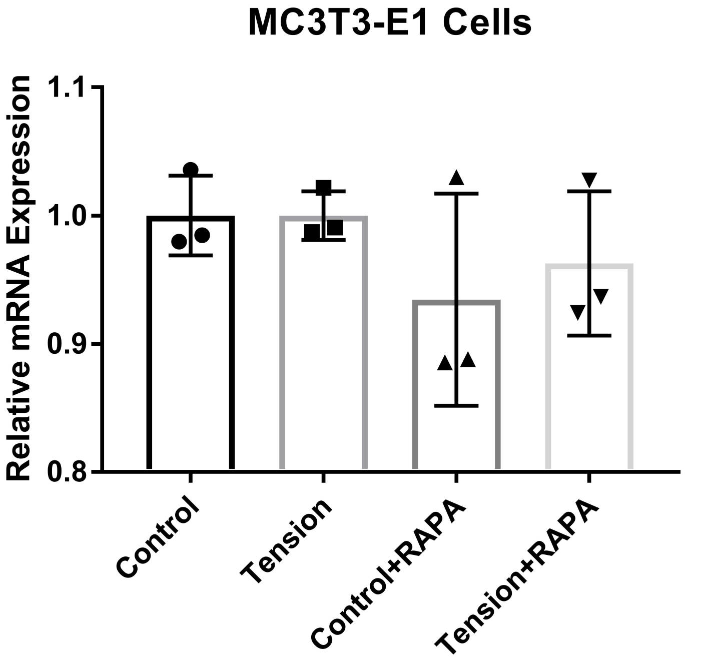

MC3T3-E1 Cells
Relative mRNA Expression
1.1
1.0
0.9
0.8
Control
Tension
Control+RAPA
Tension+RAPA

D

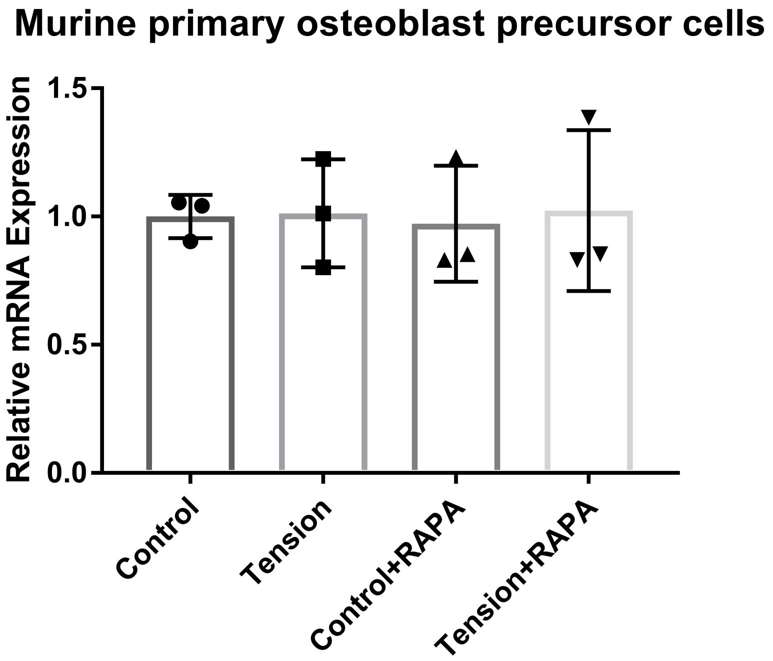

Murine primary osteoblast precursor cells
Relative mRNA Expression
1.5
1.0
0.5
0.0
Control
Tension
Control+RAPA
Tension+RAPA
