# Supplementary material for: Osteocytes Enhance Osteogenesis by Autophagy-Mediated FGF23 Secretion Under Mechanical Tension
Source: Front Cell Dev Biol. 2022 Jan 31;9:782736. doi: 10.3389/fcell.2021.782736 (PMC8841855; doi:10.3389/fcell.2021.782736)

S3 Raw images of Western Blotting

Figure 2B

1 GAP

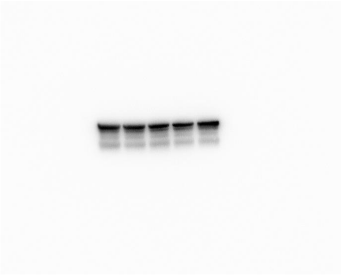

2 LC3

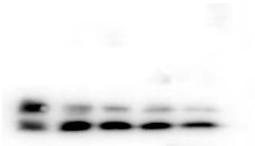

3 ATG7

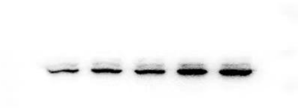

4 P62

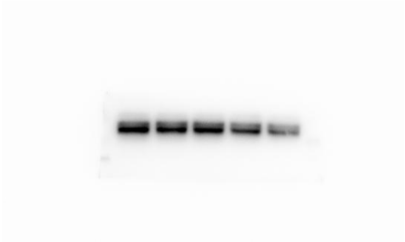

Figure 3I

1 GAP

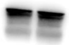

2 ALP

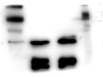

3 OPN

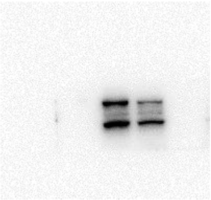

4 RUNX2

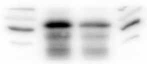

Figure 3J

1 GAP

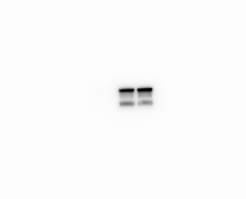

2 ALP

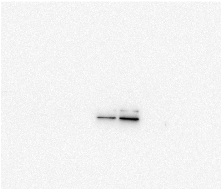

3 OPN

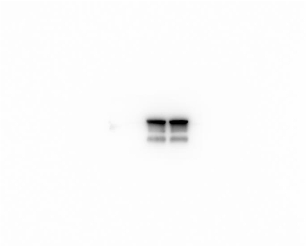

4 RUNX2

Figure 4

1 GAP

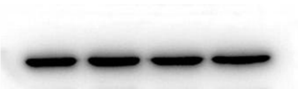

2 LC3

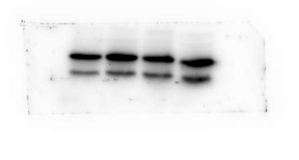

3 ATG7

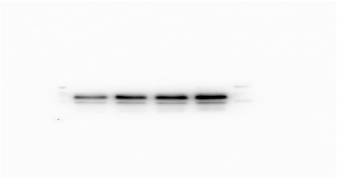

4 P62

Figure 5

1 GAP

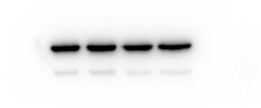

2 FGF23

Figure 6A

1 GAP

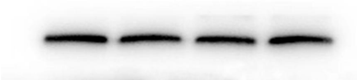

2 CARM1

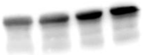

3 AMPK

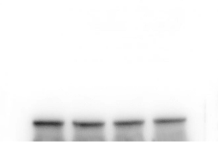

4 pAMPKa

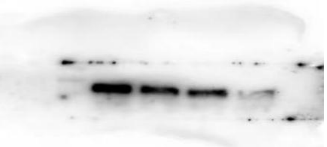

Figure 6B

1 GAP

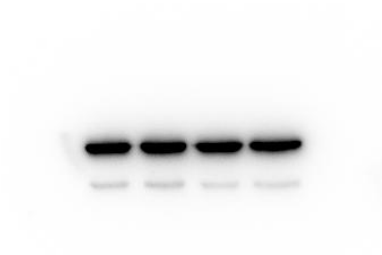

2 LC3B

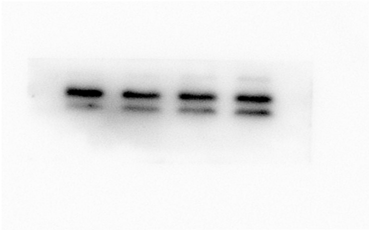

3 CARM1

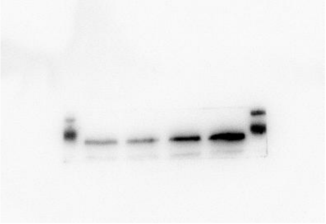

4 P62

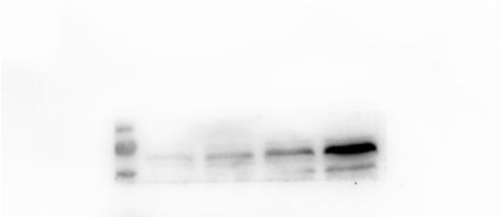

5 AMPKa

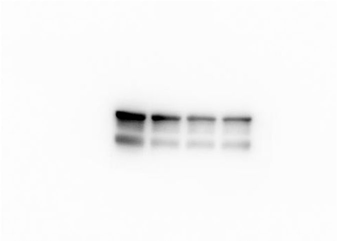

6 pAMPKa

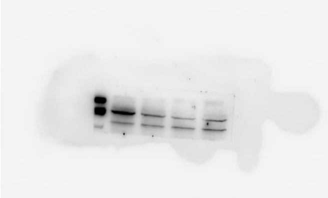

Supplement: Supplementary file 2 [file DataSheet3.PDF]
