# Supplementary material for: Osteocytes Enhance Osteogenesis by Autophagy-Mediated FGF23 Secretion Under Mechanical Tension
Source: Front Cell Dev Biol. 2022 Jan 31;9:782736. doi: 10.3389/fcell.2021.782736 (PMC8841855; doi:10.3389/fcell.2021.782736)

1. ATG4

- **Amplification Plot**

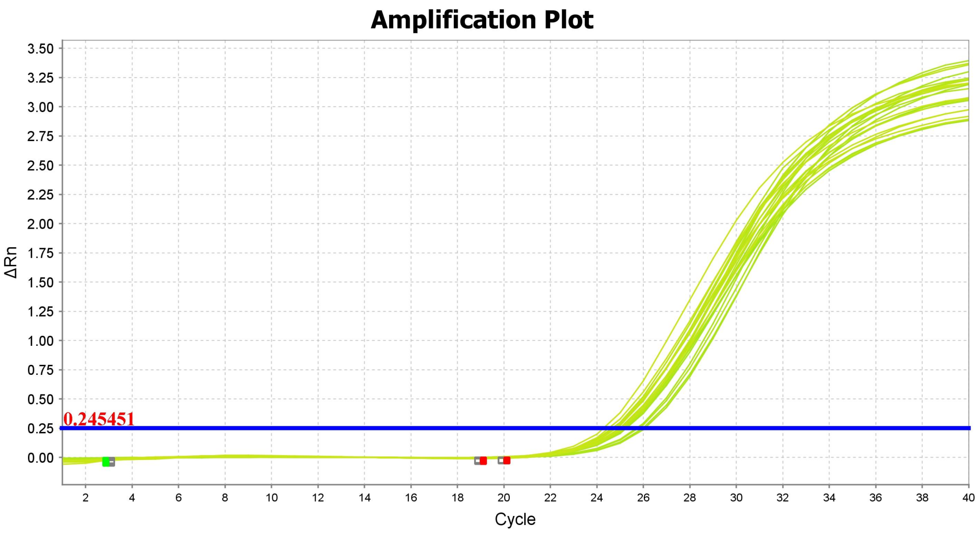

- **Melt Curve Plot**

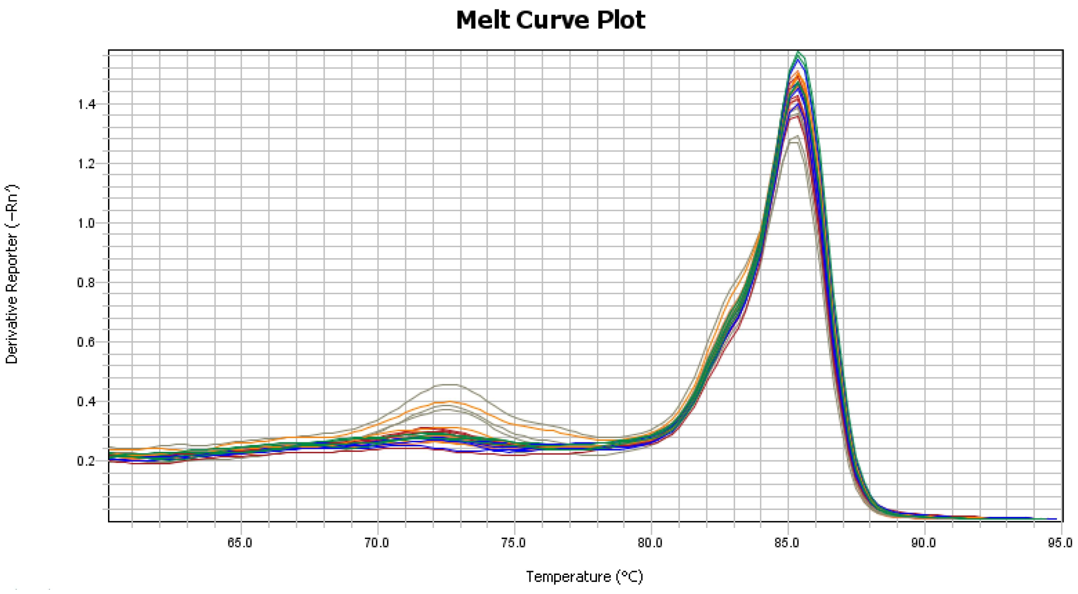

2. ATG5

- **Amplification Plot**

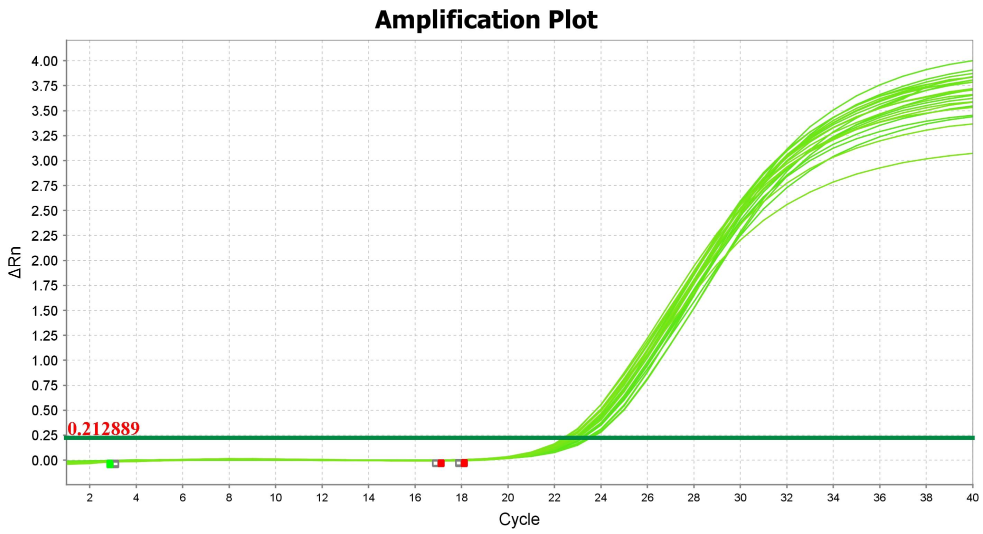

- **Melt Curve Plot**

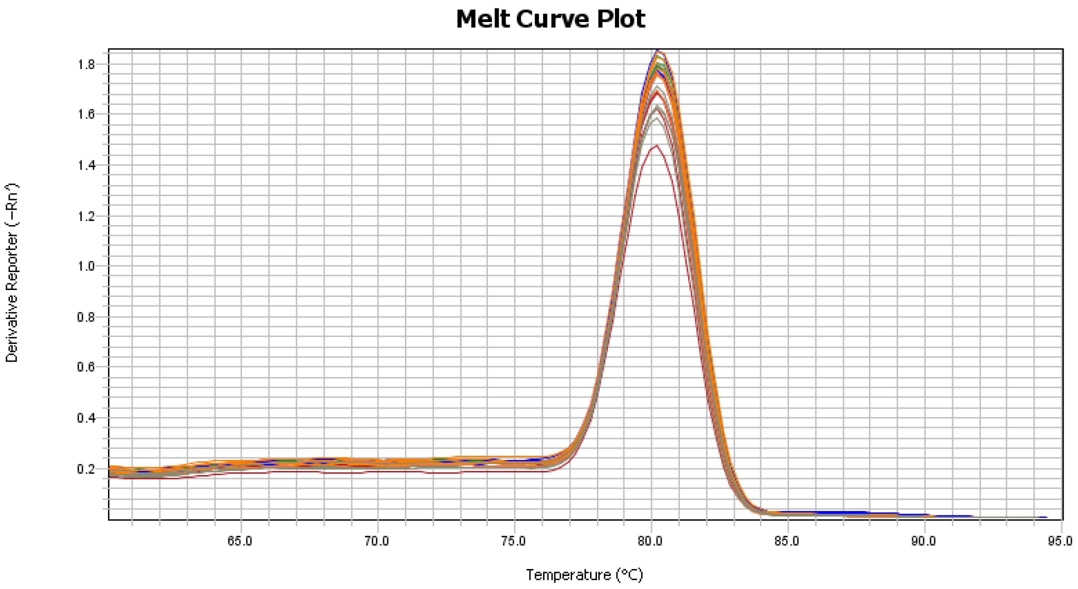

3. ATG7

- **Amplification Plot**

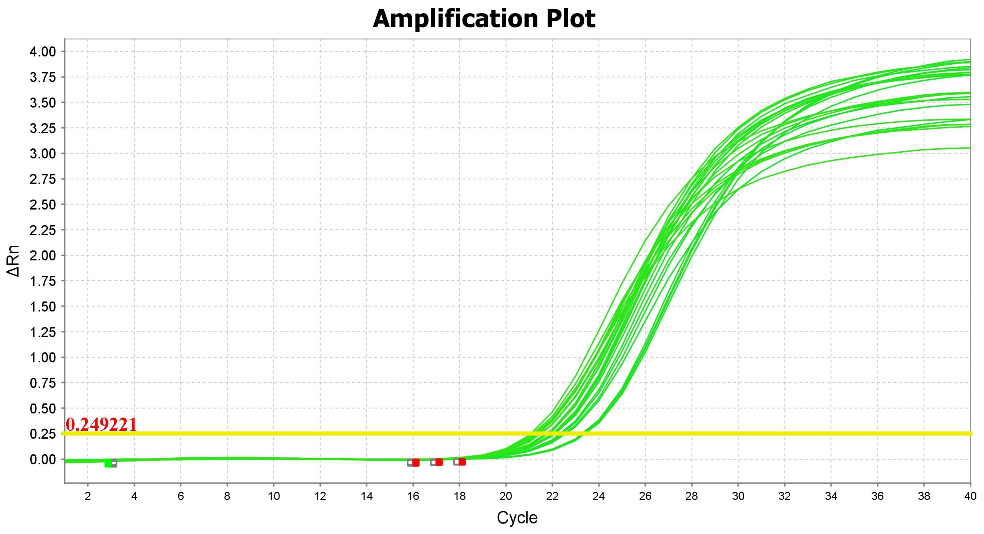

- **Melt Curve Plot**

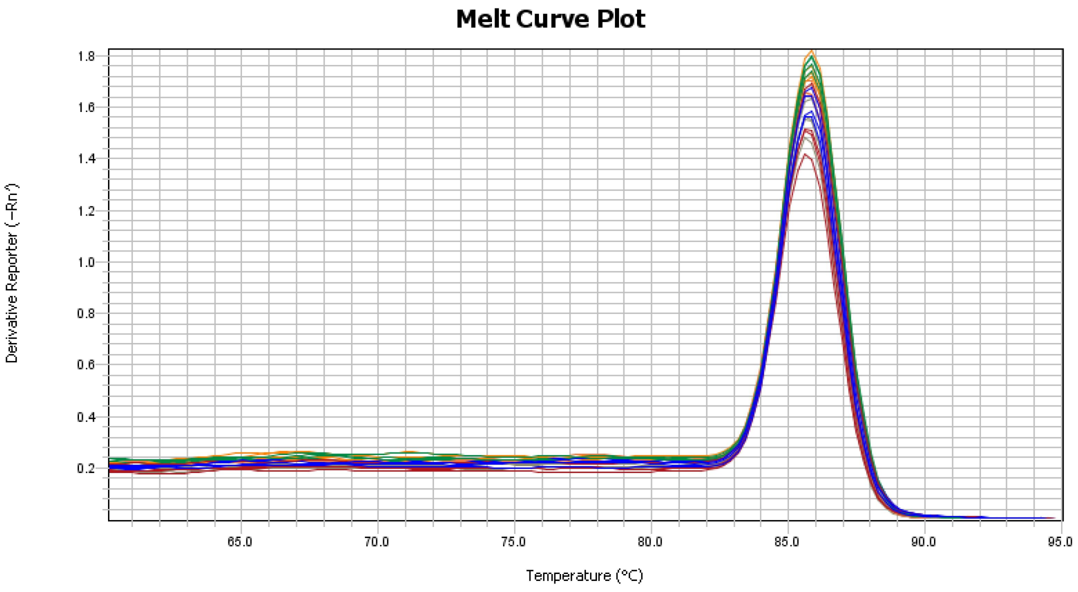

4. LC3B

- **Amplification Plot**

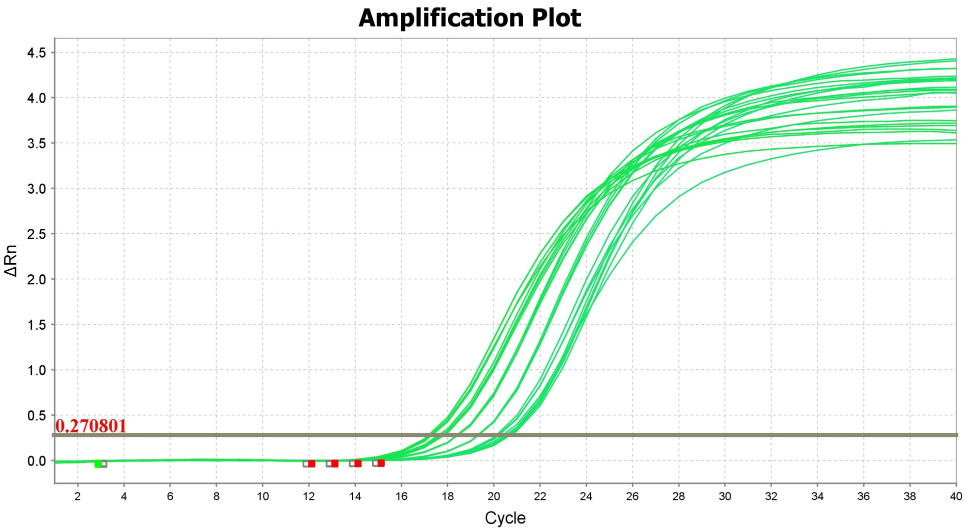

- **Melt Curve Plot**

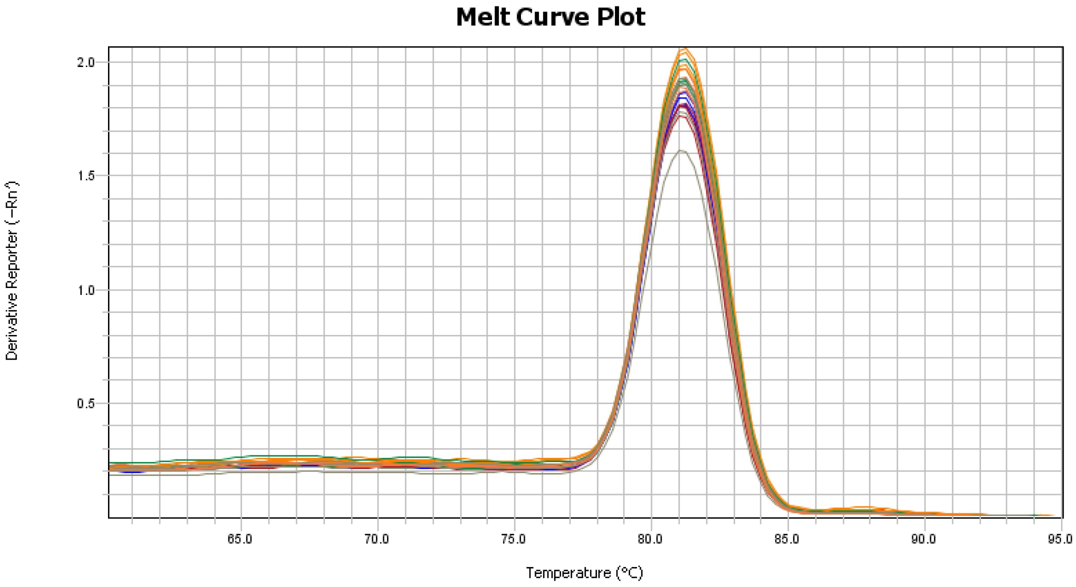

5. **P62**

- **Amplification Plot**

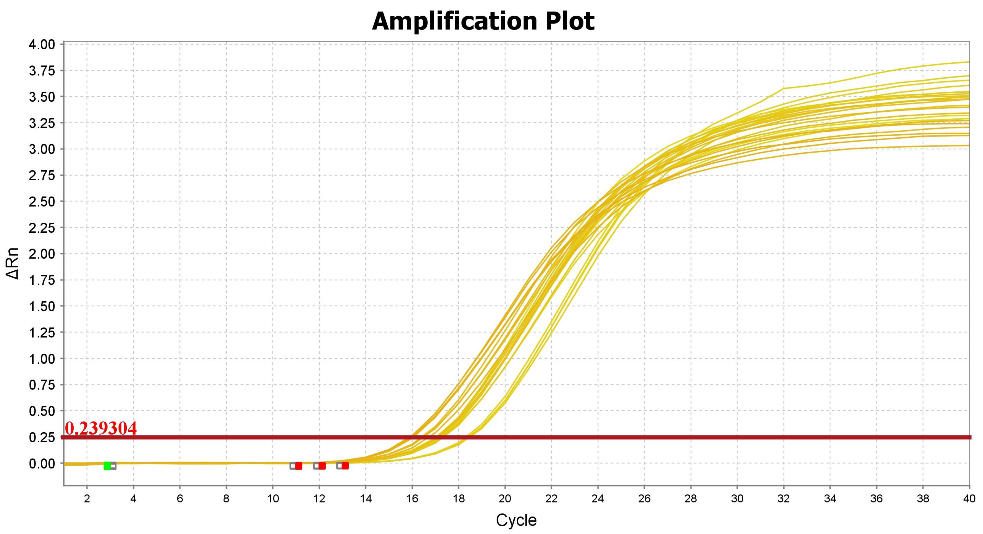

- **Melt Curve Plot**

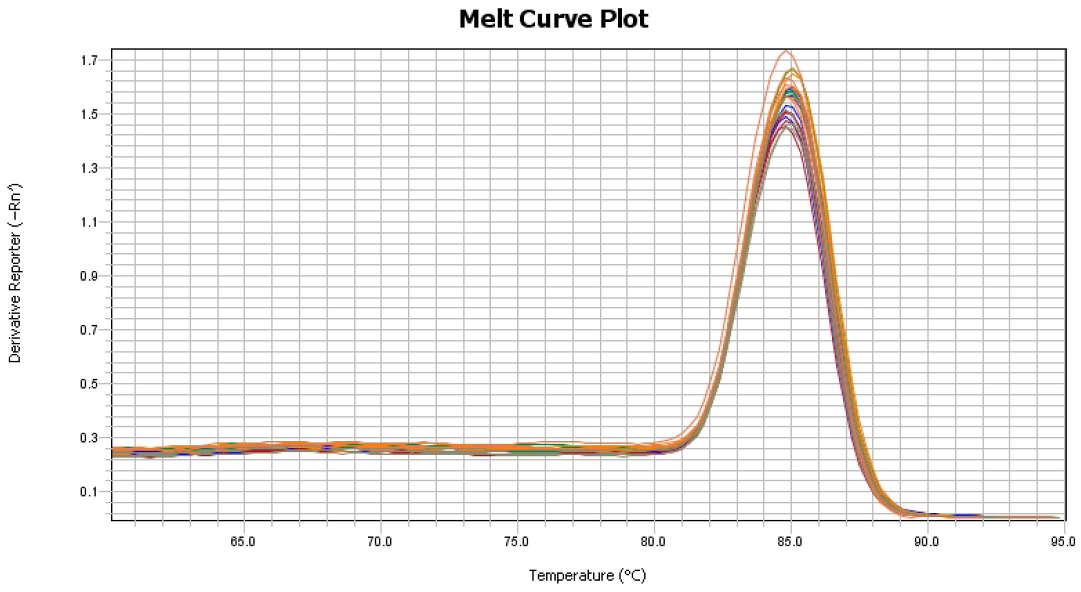

6. ULK1

- **Amplification Plot**

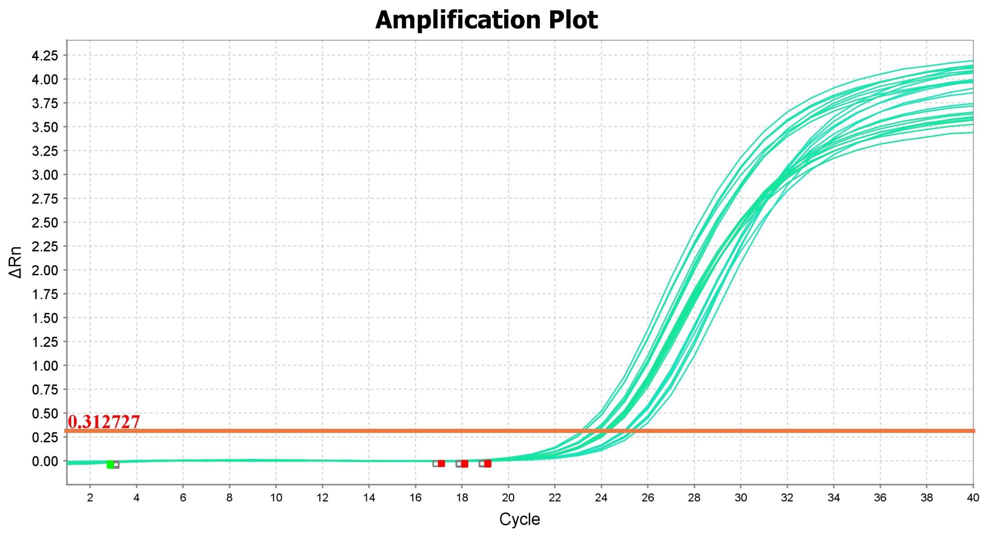

- **Melt Curve Plot**

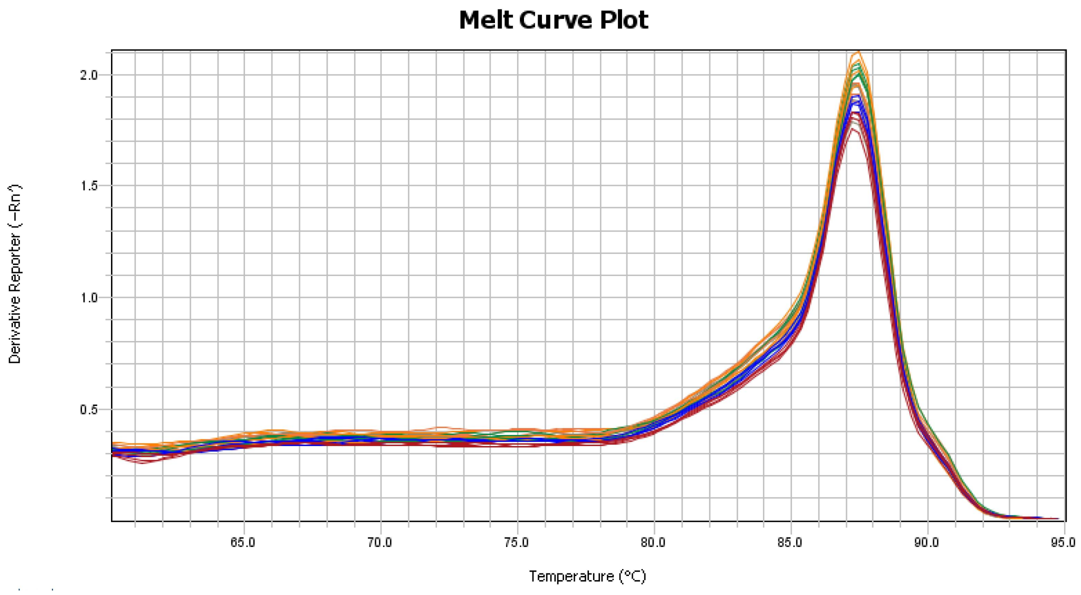

7. OCN

- **Amplification Plot**

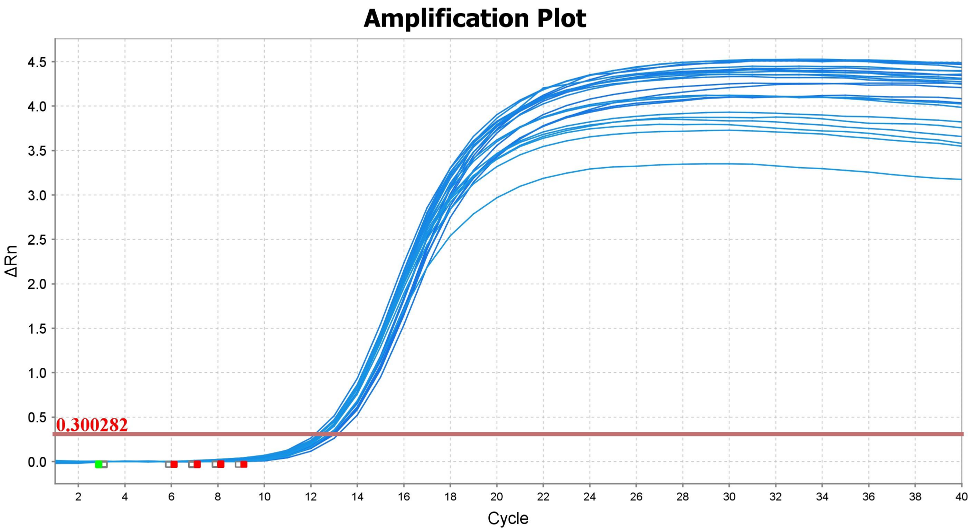

- **Melt Curve Plot**

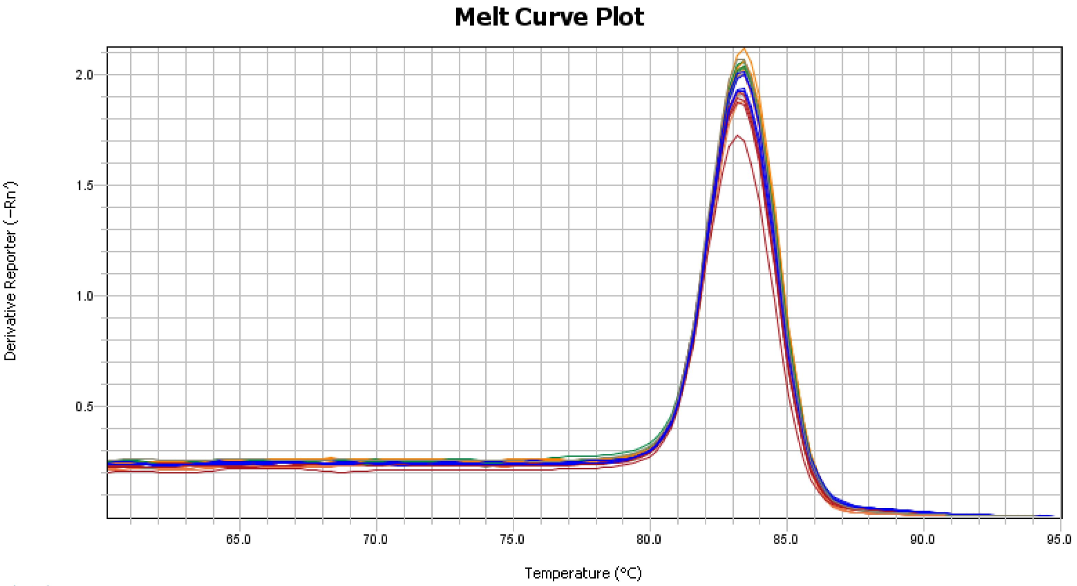

8. OPN

- **Amplification Plot**

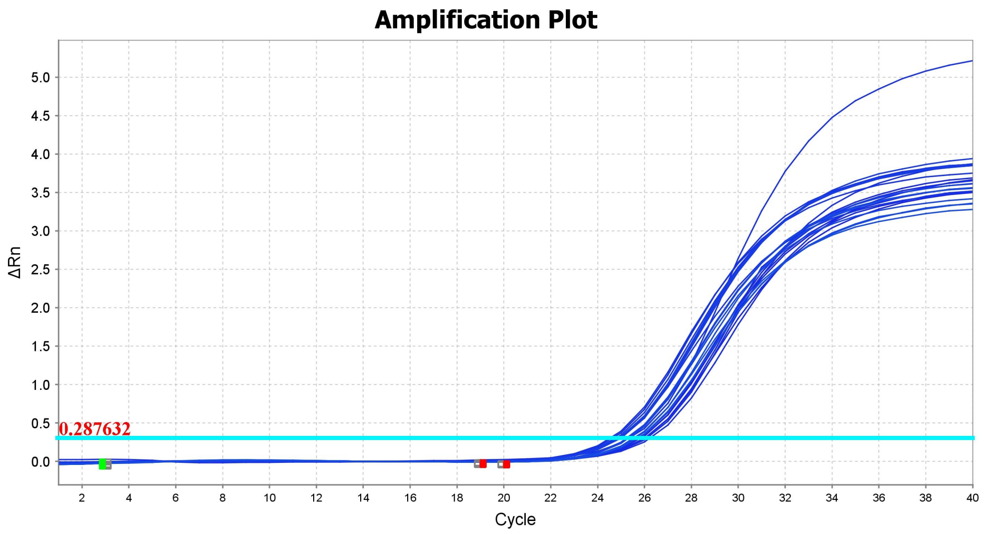

- **Melt Curve Plot**

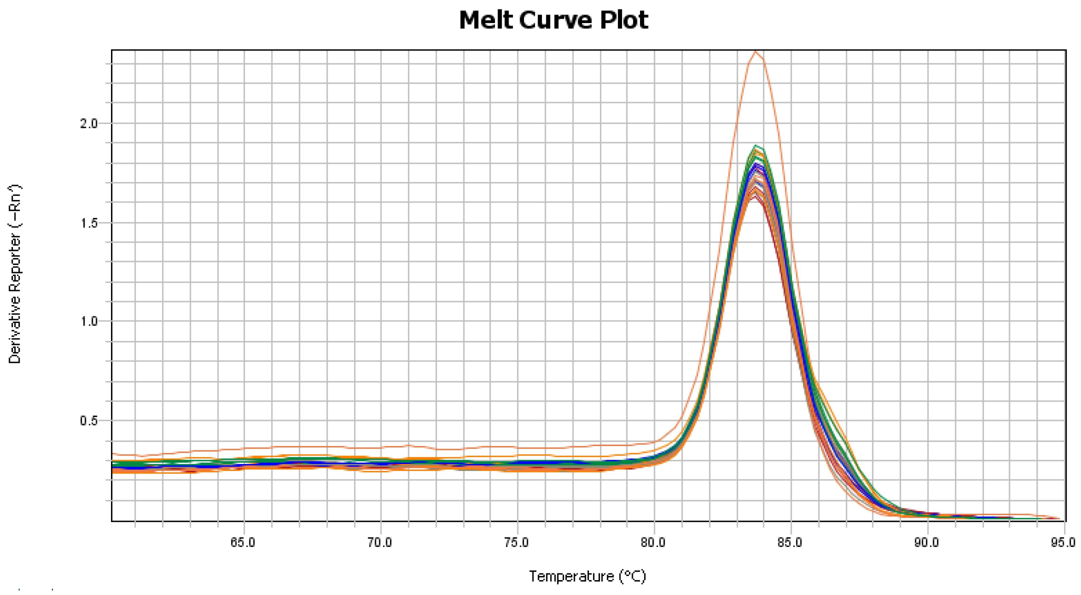

9. ALP

- **Amplification Plot**

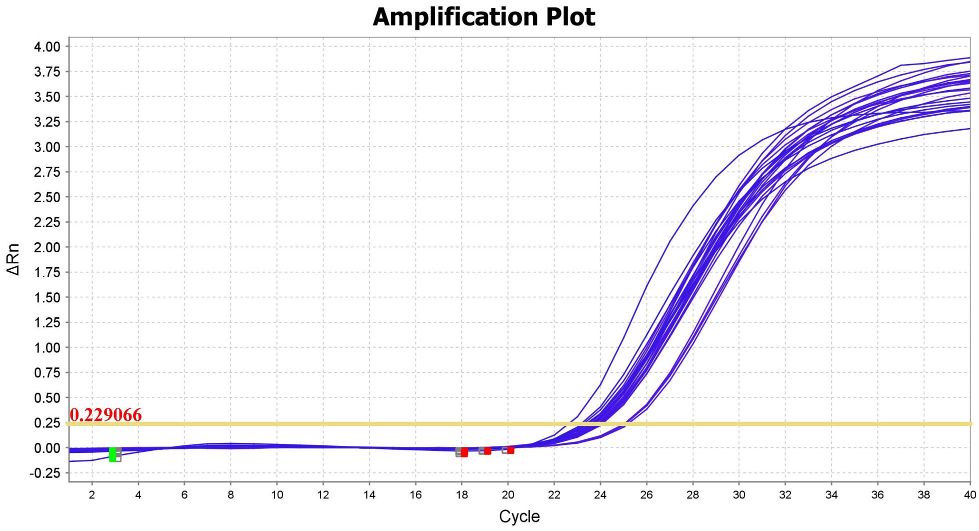

- **Melt Curve Plot**

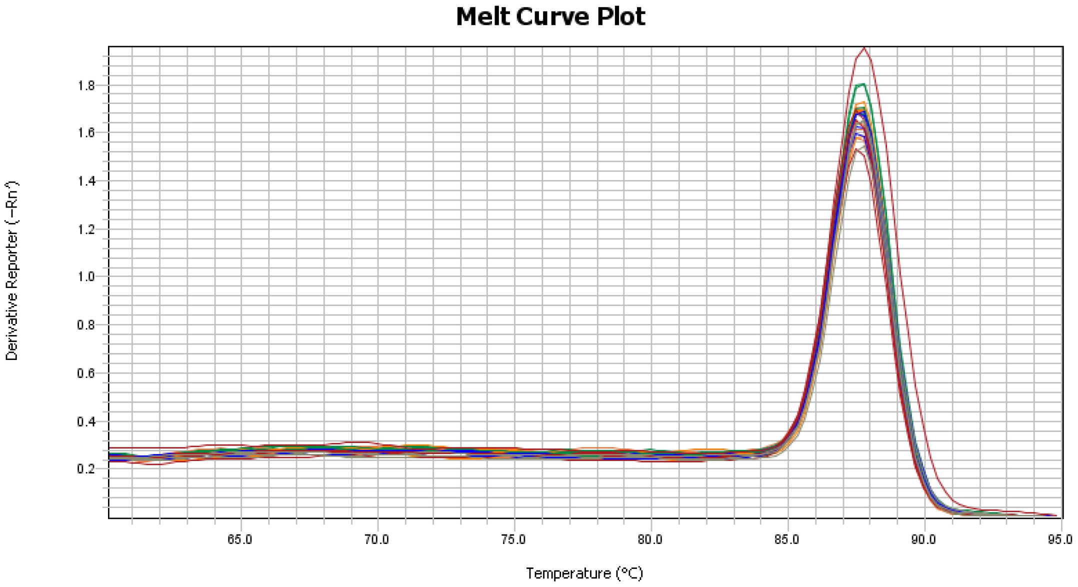

10. RUNX2

- **Amplification Plot**

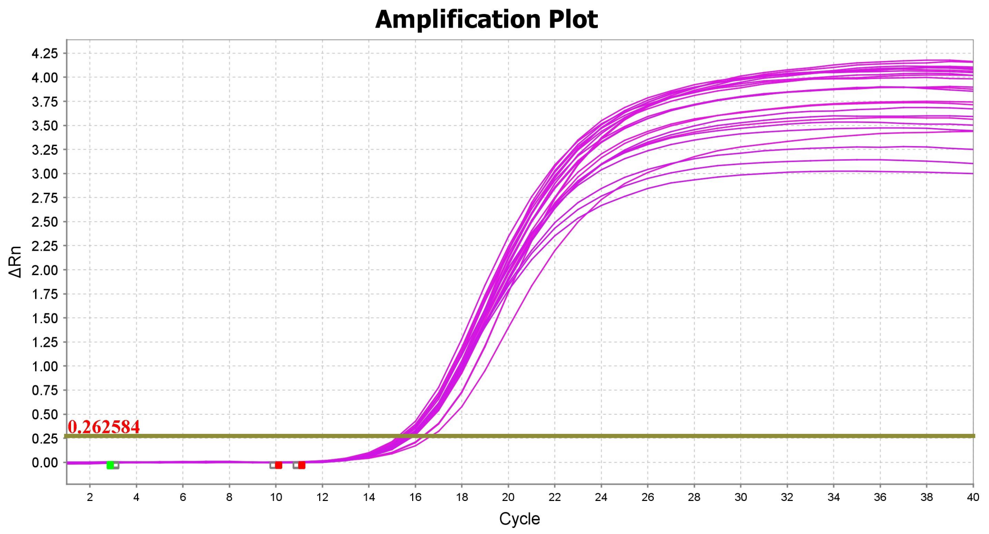

- **Melt Curve Plot**

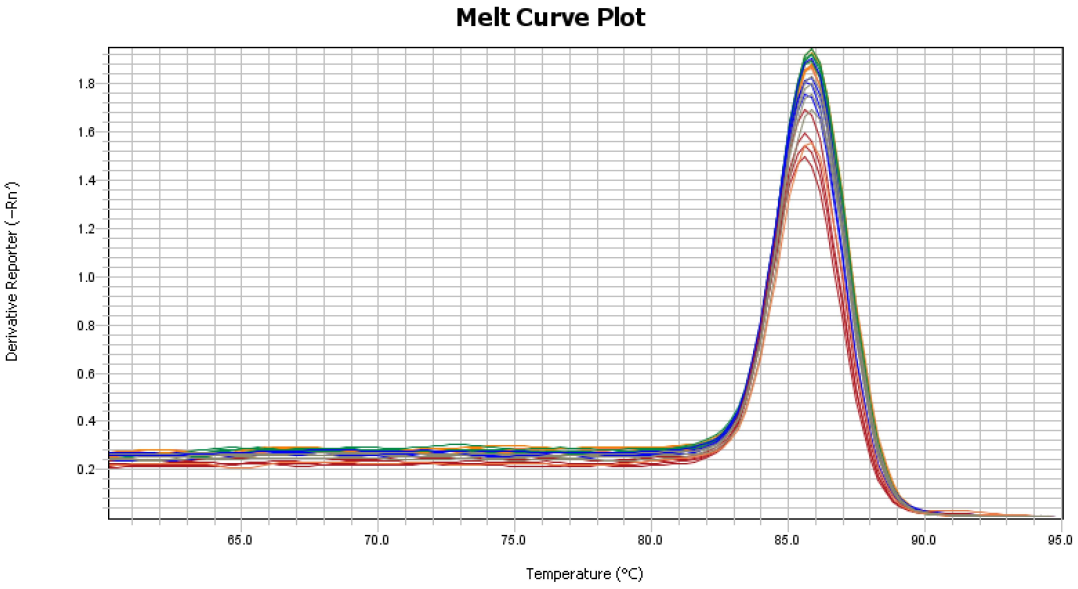

11. FGF23

- ### Amplification Plot

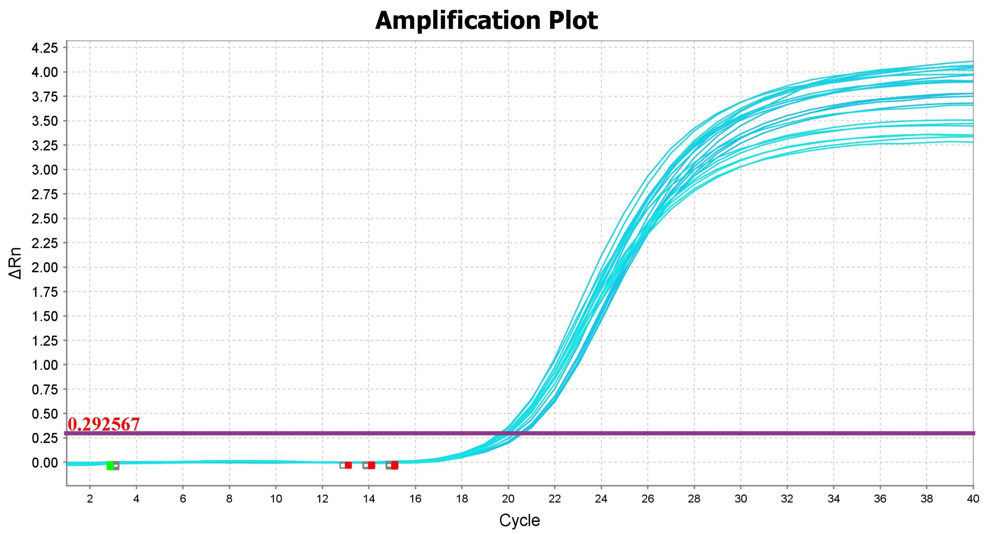

- ### Melt Curve Plot

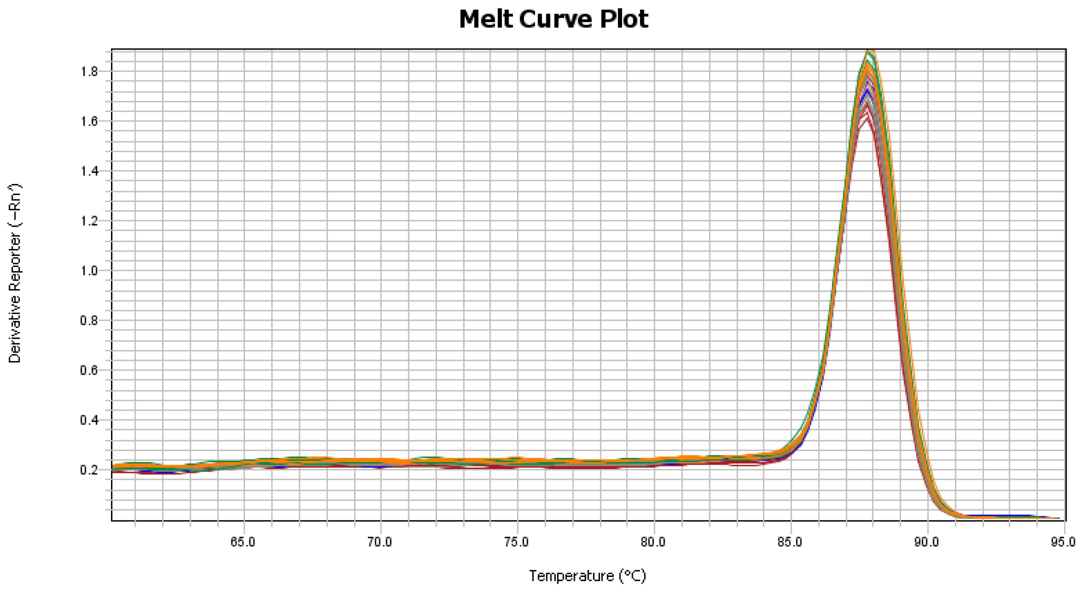

12. GAP

- **Amplification Plot**

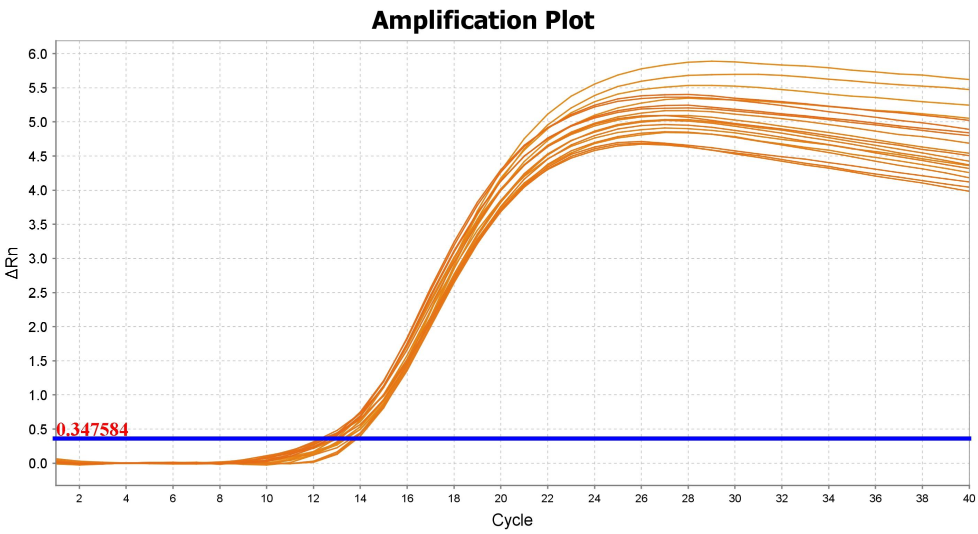

- **Melt Curve Plot**

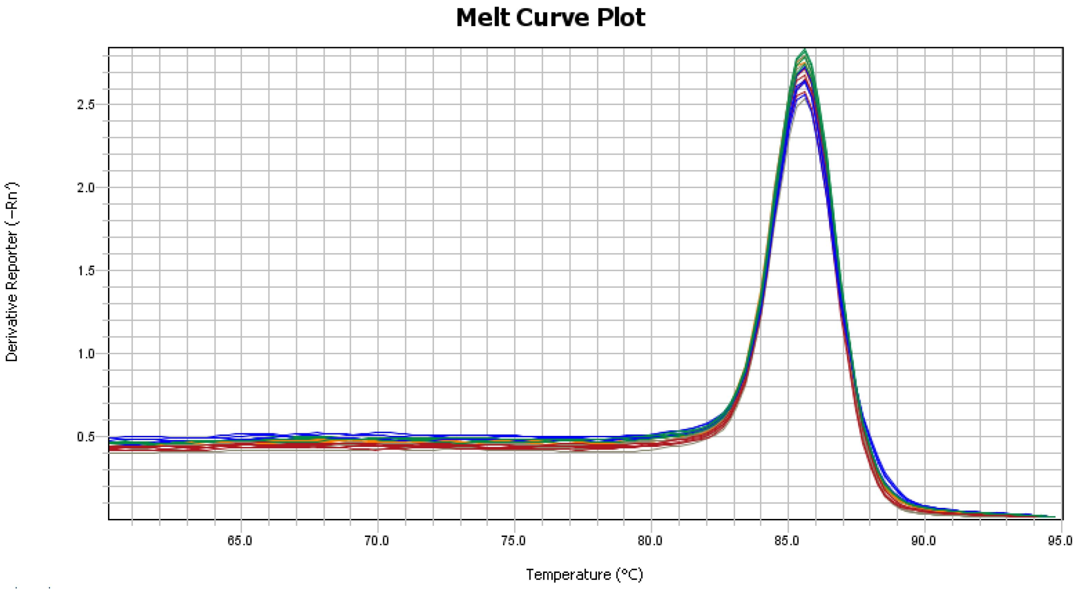

Supplement: Supplementary file 3 [file DataSheet1.PDF]
